# Characterizing Rapid Fluctuations of Resting State Functional Connectivity in Demyelinating, Neurodegenerative, and Psychiatric Conditions: From Static to Time-Varying Analysis

**DOI:** 10.3389/fnins.2019.00618

**Published:** 2019-07-10

**Authors:** Paola Valsasina, Milagros Hidalgo de la Cruz, Massimo Filippi, Maria A. Rocca

**Affiliations:** ^1^Neuroimaging Research Unit, Institute of Experimental Neurology, Division of Neuroscience, IRCCS San Raffaele Scientific Institute, Milan, Italy; ^2^Vita-Salute San Raffaele University, Milan, Italy; ^3^Neurology Unit, IRCCS San Raffaele Scientific Institute, Milan, Italy

**Keywords:** multiple sclerosis, neurodegenerative conditions, time-varying, functional connectivity, resting state, fMRI

## Abstract

Functional magnetic resonance imaging (fMRI) at resting state (RS) has been widely used to characterize the main brain networks. Functional connectivity (FC) has been mostly assessed assuming that FC is static across the whole fMRI examination. However, FC is highly variable at a very fast time-scale, as demonstrated by neurophysiological techniques. Time-varying functional connectivity (TVC) is a novel approach that allows capturing reoccurring patterns of interaction among functional brain networks. Aim of this review is to provide a description of the methods currently used to assess TVC on RS fMRI data, and to summarize the main results of studies applying TVC in healthy controls and patients with multiple sclerosis (MS). An overview of the main results obtained in neurodegenerative and psychiatric conditions is also provided. The most popular TVC approach is based on the so-called “sliding windows,” in which the RS fMRI acquisition is divided in small temporal segments (windows). A window of fixed length is shifted over RS fMRI time courses, and data within each window are used to calculate FC and its variability over time. Sliding windows can be combined with clustering techniques to identify recurring FC states or used to assess global TVC properties of large-scale functional networks or specific brain regions. TVC studies have used heterogeneous methodologies so far. Despite this, similar results have been obtained across investigations. In healthy subjects, the default-mode network (DMN) exhibited the highest degree of connectivity dynamism. In MS patients, abnormal global TVC properties and TVC strengths were found mainly in sensorimotor, DMN and salience networks, and were associated with more severe structural MRI damage and with more severe physical and cognitive disability. Conversely, abnormal TVC measures of the temporal network were correlated with better cognitive performances and less severe fatigue. In patients with neurodegenerative and psychiatric conditions, TVC abnormalities of the DMN, attention and executive networks were associated to more severe clinical manifestations. TVC helps to provide novel insights into fundamental properties of functional networks, and improves the understanding of brain reorganization mechanisms. Future technical advances might help to clarify TVC association with disease prognosis and response to treatment.

## Introduction

The human brain at resting state (RS) exhibits highly structured spontaneous fluctuations in functional magnetic resonance imaging (fMRI) data, which reflect the underlying network architecture (Biswal et al., 2010). RS functional connectivity (FC) captures the temporal associations between such fluctuations, and has been successfully used to characterize the main networks of the brain and map abnormalities of functional network architecture occurring in different neurological conditions. In healthy controls, RS FC strength was found to be associated to age, with RS fluctuations being strongest in adulthood and lowest in children and elderly (Mak et al., 2017). A dependency of connectivity from sex (Biswal et al., 2010; Mak et al., 2017), as well as from cognitive, emotional, and behavioral variables was also detected (Kelly et al., 2012).

Multiple sclerosis (MS) is an inflammatory and neurodegenerative disease of the central nervous system leading to a progressive increase over time of clinical disability and cognitive impairment (Filippi et al., 2017, 2018). Reorganization of brain functional networks in MS has been shown from the first RS fMRI studies (Lowe et al., 2002, 2008; Rocca et al., 2010; Roosendaal et al., 2010), which is thought to limit the clinical consequences of widespread tissue damage (Filippi et al., 2013a; Sbardella et al., 2015). Cortical reorganization has been demonstrated to be variable across the different stages of the disease, and a progressive exhaustion or inefficiency of the adaptive properties of the cerebral cortex is likely to be among the factors responsible for the worsening of clinical disability (Rocca et al., 2010, 2018; Roosendaal et al., 2010; Loitfelder et al., 2011). In neurodegenerative conditions, RS FC studies showed a progressive and gradual spreading of connectivity changes from a target brain network, reflecting specific behavioral and cognitive dysfunctions (Zhou et al., 2017). In psychiatric diseases, disruption of fronto-parietal network connectivity seems to be the common fingerprint across distinct forms of pathology (Baker et al., 2019).

However, current understanding of the role of functional abnormalities in neurological and psychiatric disorders is still incomplete, mostly due to inconsistencies in the findings from several studies. Specifically, in MS some investigations found trends toward lower RS FC vs. healthy controls in the default-mode (Rocca et al., 2010, 2012, 2018; Bonavita et al., 2011), sensorimotor (Rocca et al., 2018) and subcortical (Liu et al., 2011; Rocca et al., 2018) networks, while in other studies the opposite trends were observed (Roosendaal et al., 2010; Tona et al., 2014; Schoonheim et al., 2015). Similarly, even if RS FC abnormalities were principally located in the core regions hit by pathology, a certain variability of brain areas involved by RS FC changes was detected in neurodegenerative and psychiatric conditions (Busatto, 2013; Weiner et al., 2017).

The wide spectrum of clinical characteristics of MS patients has been considered as one of the main causes for the discrepancies described in RS fMRI literature (Filippi et al., 2013a; Sbardella et al., 2015). However, technical factors might also bias connectivity estimation, including scanner-related signal instabilities, an inappropriate control of confounding covariates, and the application of analysis methods based on inaccurate assumptions.

For instance, one of the main assumptions of classical RS FC assessment methods is that connectivity is static across the entire fMRI examination, e.g., it can be assessed by calculating the mean correlation between whole-length RS fMRI time series (Biswal et al., 2010). However, as widely evident by neurophysiological techniques, brain FC is highly variable at a very fast time-scale. The functioning human brain during any state of wakefulness repeatedly changes between different combinations of cognitive, sensorimotor, attentional, emotional, auditory, and visual-related tasks. Notably, the majority of brain regions experience continuous functional changes even during sleep (Tagliazucchi and van Someren, 2017). Thus, studying time-varying RS FC patterns is likely to shed light not only on physiological processes occurring in healthy subjects, but also to understand clinical manifestations of different neurological and psychiatric conditions. In fact, clinical symptoms associated to these diseases are likely to depend not only from damage to specific brain regions, but also from delayed (or abnormal) communication between brain areas. The study of the temporal reconfigurations of FC occurring within RS fMRI sessions has been defined as *time-varying functional connectivity (TVC)* (Hutchison et al., 2013; Calhoun et al., 2014; Preti et al., 2017).

The main goal of this review is to summarize the main results obtained using TVC in healthy and diseased populations. A particular focus is given to studies of patients with MS; however, the main findings of investigations performed in neurodegenerative and psychiatric conditions are also reported. The review is structured as follows: in section Methods Used to Assess Time-Varying Functional Connectivity, we present the main approaches developed to investigate TVC using fMRI data, with a main emphasis on the methods applied to study MS patients. Then, we summarize the results obtained applying these methods in healthy controls (section Application of TVC to Healthy Subjects) and in patients with MS (section Application of TVC Techniques to MS). An overview of the results derived from other neurological and psychiatric conditions is also given (section Application of Time-Varying FC Techniques to Psychiatric and Other Neurological Diseases). In the final part (section Current Limitations and Future Directions), current TVC methodological limitations are discussed and possible future developments are presented.

## Methods Used to Assess Time-Varying Functional Connectivity

Several analysis strategies have been applied so far to quantify temporal variations of blood oxygenation level dependent (BOLD) signal fluctuations (Hutchison et al., 2013; Preti et al., 2017). Some strategies aim at capturing variations in inter-regional associations between pairs of brain areas (Sakoglu et al., 2010; Allen et al., 2014), while others try to detect changing patterns of temporal synchrony at a multivariate level, e.g., considering all brain regions at once (Tagliazucchi et al., 2012; Liu and Duyn, 2013). One of the most popular methods for TVC analysis, which is based on the use of the so-called “sliding windows” (Sakoglu et al., 2010; Allen et al., 2014), belongs to the first category, since it relies on the calculation of a series of pairwise correlation coefficients over small shifting segments of fMRI time series.

Despite the great variability of available pipelines, TVC analysis usually requires the performance of the following steps: (1) selection of a set of regions of interest (ROIs) in the brain; (2) assessment of time-varying correlations among the selected ROIs; and (3) extraction of features quantifying connectivity changes over time, as described in details in the next paragraphs.

### Selection of Regions of Interest for TVC Analysis

It is important to properly identify the ROIs (which may be areas of the brain, or even entire functional networks) that will be included in TVC analysis. Several factors can influence the choice of ROIs: spatial resolution, the use of *a priori* hypotheses or data-driven strategies, and the rationale of the experiment, which may focus on selected functional circuits or on the whole brain.

**Figure 1:**
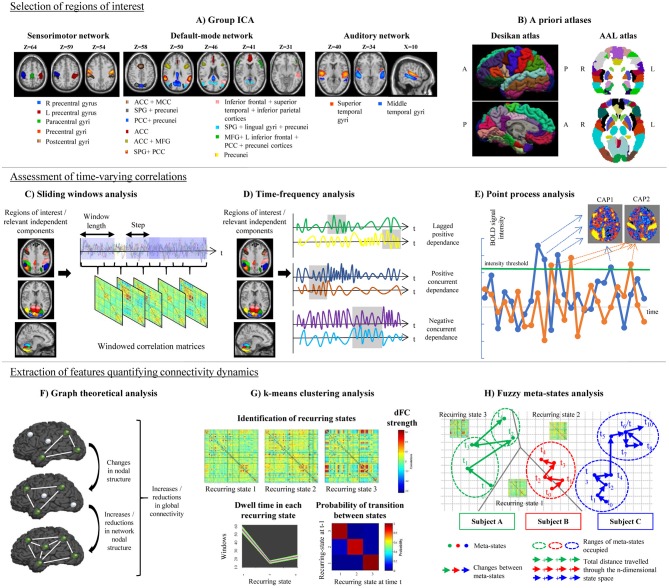
Schematic representation of the post-processing steps used in the assessment of time-varying functional connectivity (TVC). Top row: selection of regions of interest for TVC analysis, which can be done using data-driven approaches (e.g., independent component analysis, **A**) or using a priori atlases **(B)**. Middle row: assessment of time-varying correlations between fMRI time series. The most popular approach consists in using a sliding-window analysis **(C)**; alternative approaches, such as time-frequency analysis **(D)** or point-process analysis **(E)** have been also proposed. Bottom row: extraction of features quantifying connectivity changes over time, which can be done using several techniques, such as graph theory **(F)**, k-means clustering to estimate recurring TVC states **(G)**, or fuzzy meta-state analysis **(H)**. ICA, independent component analysis; AAL, automatic anatomical labeling; ACC, anterior cingulate cortex; CAP, co-activation pattern; MCC, middle cingulate cortex; PCC, posterior cingulate cortex; SPG, superior parietal gyrus; MFG, middle frontal gyrus; R, right; L, left.

The large majority of studies assessing TVC in MS patients mainly relied on the use of *a priori* atlases, such as the Automatic Anatomical Labeling (AAL) (Tzourio-Mazoyer et al., 2002) or the Desikan (Desikan et al., 2006) cortical atlas (Leonardi et al., 2013; Lin et al., 2018; van Geest et al., 2018a,b). Some studies built *ad-hoc* ROIs centered in critical nodes of large-scale brain networks (Bosma et al., 2018). However, a widely used approach in previous literature consists in a ROI data-driven selection through independent component analysis (ICA; Rocca et al., 2010, 2019; Sakoglu et al., 2010; Filippi et al., 2013b; Allen et al., 2014; Damaraju et al., 2014; Yang et al., 2014; Zalesky et al., 2014; Bisecco et al., 2018; Castellazzi et al., 2018; d'Ambrosio et al., 2019) (Figure 1). The broad application of ICA in previous TVC studies can be explained by the flexibility of this approach, which allows to extract ROIs at different spatial resolution according to ICA dimensionality, to perfectly fit the data (avoiding non-linear registrations with *a priori* atlases, which may be challenging in diseased populations) and to reduce the impact of physiological and motion-related noise.

Since ROIs identified by “static” *a priori* atlases may not reflect significant connectivity variations occurring within brain regions at short time scales (Ryyppo et al., 2018), recent studies have suggested that incorporating information of time-varying connectivity between neighboring voxels to parcellate the brain may improve accuracy of TVC analyses (Preti and Van De Ville, 2017; Ryyppo et al., 2018).

### Assessment of Time-Varying Correlations Among Brain Regions

#### Sliding Window Analysis

The most popular strategy used to examine time-varying correlations between RS fMRI time series relies on the use of sliding windows (Sakoglu et al., 2010; Allen et al., 2014). In this approach, a time window of fixed length is selected, and correlations between pairs of fMRI time series are calculated using data within that window. Then, the window is shifted in time by a certain number of time points, and correlations are re-assessed on the new data. This procedure results in a series of pair-wise correlation matrices that describe the time-resolved behavior of connectivity over the entire duration of the fMRI experiment (Allen et al., 2014; Figure 1).

The choice of an appropriate length for sliding windows is crucial: too short time segments may introduce spurious fluctuations associated with intrinsic fMRI signal instability, while with increased window size TVC estimation may become too similar to the classic static FC (Leonardi and Van De Ville, 2015; Preti et al., 2017). Different validation analyses recommended to set window length around 30–60 s (or the equivalent time expressed as repetition times, TRs), demonstrating consistent reproducibility of the obtained results (Allen et al., 2014; Damaraju et al., 2014; Rashid et al., 2014, 2016; Zalesky et al., 2014; Leonardi and Van De Ville, 2015; Qin et al., 2015; Zalesky and Breakspear, 2015; Choe et al., 2017; Zhang C. et al., 2018).

Once sliding windows correlation matrices have been produced, different strategies can be applied to extract features describing connectivity reorganization through time inside the data (Leonardi et al., 2013; Allen et al., 2014; Miller et al., 2016), as described in details in section Extraction of Features Quantifying Time-Varying Connectivity.

#### Beyond Sliding-Window Analysis

A variety of approaches alternative to sliding windows have been developed to quantify TVC in fMRI data (Preti et al., 2017). For instance, time-frequency decomposition has been used to represent correlations between two fMRI time series in the joint time and frequency domain (Chang and Glover, 2010; Yaesoubi et al., 2015a; Figure 1). Point-process analysis allowed to detect recurring patterns of co-activation between brain regions from a small fraction of the total scans of a RS fMRI experiment (Tagliazucchi et al., 2012; Liu and Duyn, 2013). Phase coherence connectivity has been proposed to calculate RS FC at each recorded fMRI time point (Deco and Kringelbach, 2016).

In MS studies, two alternative methods to sliding windows have been applied. One study (Bosma et al., 2018) used dynamic conditional correlations (DCC) to quantify TVC. DCC were originally proposed to study fluctuations of financial time series (Engle, 2002) and subsequently adapted to neuroimaging data to quantify time-varying variances and correlations between multivariate RS fMRI time series (Lindquist et al., 2014). DCC overcome some limitations intrinsic to sliding-window techniques, since they do not depend from any arbitrary window length and do not give the same weight to all time points within the window, ignoring older observations. Moreover, DCC are not easily confused by changes of correlation occurring in fMRI time series merely due to random noise (Lindquist et al., 2014).

Another study (Zhou et al., 2016) quantified connectivity reorganization over time using brain entropy (BEN). Entropy is a statistical and physical index that measures irregularity of a time-varying system (Sandler, 2006). In RS fMRI data, voxel-wise assessments of entropy were performed by calculating sample entropy, defined as the negative logarithm of the probability that if two time series of length *m* have a correlation < *r*, then two time series of length *m*+*1* also have a correlation < *r*. A higher entropy indicates increased randomness of a system, meaning that the time-varying system activity is less predictable and less organized (Wang et al., 2014).

### Extraction of Features Quantifying Time-Varying Connectivity

Sliding-window (or alternative) techniques produce a large amount of correlation data, calculated on several time segments. Some features have then to be extracted from this big data mass, to summarize to what extent functional relationships reorganize through time. The simplest summary TVC statistic is standard deviation (or variance) of sliding-window correlation time series (Sakoglu et al., 2010; Choe et al., 2017) or of DCC time series (Lindquist et al., 2014; Bosma et al., 2018). The mean TVC (Huang et al., 2019) or the sum of absolute differences in pair-wise connectivity between consecutive windows have also been used as a summary TVC measure (van Geest et al., 2018a,b). Another interesting metric assessing temporal variability of BOLD fluctuations is the so-called amplitude of low-frequency fluctuations functional connectivity (ALFF-FC) (Shen et al., 2016), which sums up the spectral content of low-frequency RS fluctuations through consecutive sliding windows.

Flexibility metrics quantifying time-varying global and regional network properties were also calculated using a graph theory framework (Lin et al., 2018), as described in section Graph Theoretical Analysis. More complex strategies rely on the identification of connectivity patterns that reoccur over time during the course of the experiment. Reoccurring RS FC patterns, often called “states,” can be determined using clustering techniques (Allen et al., 2014), principal component analysis (Leonardi et al., 2013) or tensor decomposition (Mokhtari et al., 2018a), as detailed in section Definition of Reoccurring Connectivity States. Finally, approaches overcoming a rigid data decomposition into “fixed” connectivity states have been recently proposed (Miller et al., 2016), as described in detail in section Fuzzy Meta-State Analysis.

#### Graph Theoretical Analysis

Graph theory analysis can be applied to series of matrices derived from sliding-window analysis (Figure 1). Besides the classical network metrics (Rubinov and Sporns, 2010), which can be quantified as a function of time (Fukushima and Sporns, 2018), more specific metrics can be used to assess time-varying network structure. For instance, network power measures the summed values of TVC pairs in all windows, and density estimates how dense, on average, connections are over time. Specific time-resolved network features include network variation, which describes how different are connectivity values between two adjacent windows, flexibility of homologous, non-homologous and intra-hemispheric connections, which quantify connectivity differences between two consecutive windows for the specified type of connections (Lin et al., 2018), or the Fiedler value, which summarizes how well-connected a network is (Cai J. et al., 2018). Recently, novel approaches have been proposed to improve modeling of brain network TVC using graph theory (Khambhati et al., 2018). Such modeling strategies aim to assess time-varying patterns of connectivity (e.g., dynamic community detection or non-negative matrix factorization), time-varying patterns of activity, or a combination of both. A detailed review of these methods is reported in Khambhati et al. (2018).

#### Definition of Reoccurring Connectivity States

One of the most diffuse approaches used to identify reoccurring FC states from sliding-window matrices is based on hard-clustering algorithms (Preti et al., 2017), such as the k-means algorithm (Allen et al., 2014). In this approach, data are partitioned into different connectivity states by maximizing a cluster validity index, which describes the between-cluster/within-cluster distance ratio. In this way, identified recurring connectivity states have a minimal degree of overlap (Allen et al., 2014). The amount of time spent in each recurring state (dwell time) and the number of transitions between states can be calculated and compared between groups (Figure 1). Between-group comparisons can be also performed on pair-wise TVC strengths within each detected state (Allen et al., 2014).

Other ways to identify FC states from sliding-window data rely on principal component analysis (PCA) (Leonardi et al., 2013) or tensor decomposition (Mokhtari et al., 2018a). PCA is able to decompose sliding-window matrices into patterns of correlated connectivities (called “eigenconnectivities”) between brain regions. Each eigenconnectivity pattern is characterized by a “contribution” (which can be thought as the equivalent of dwell times for k-means clustering analysis), which varies over time across subjects. Between-group comparisons of such contributions may allow to characterize TVC abnormalities in patients' populations (Leonardi et al., 2013). Similarly, tensor decomposition (Mokhtari et al., 2018a) is able to decompose sliding-window connectivity matrices in a set of components, each with an associated weight, which explain the majority of data content.

#### Fuzzy Meta-State Analysis

In hard-clustering analysis, windowed correlation matrices are forced to fit into determined TVC recurring states. However, the existence of just one state at each time point may be a too rigid assumption. A more flexible approach is to consider the possibility that multiple states might be represented to varying degrees at the same time point. The contribution of each state for a specific time is characterized by a vector that is called a “meta-state” (Miller et al., 2016). Four different measures of neural reorganization over time can be associated to such meta-states and can be calculated for each study subject: (1) the total number of distinct meta-states that a subject assumes during the experiment; (2) the number of changes between distinct meta-states; (3) the range of meta-states occupied in the n-dimensional meta-state space during the entire RS fMRI experiment; and (4) the total distance traveled in the n-dimensional state space (Figure 1).

## Application of TVC to Healthy Subjects

### Main TVC Findings in Healthy Subjects

The results of the main studies assessing TVC in healthy controls are summarized in Table 1.

**Table 1:** Summary of studies assessing time-varying resting state functional connectivity in healthy subjects and simulated data.

**Study**	**RS fMRI acquisition parameters^**Ω**^**	**TVC analysis approach^**∧**^**	**Study subjects^θ^**	**Main findings**
Allen et al. (2014)	Siemens Trio 3T 152 volumes TR = 2 s	1. Group ICA decomposition in 50 relevant independent components of interest, classified into 7 different functional networks 2. Sliding-window analysis, window length = 22 TRs (44 s), steps = 1 TR (2 s). 3. k-means clustering (7 recurring states)	405 healthy adults 200 females (49.4%) mean age = 21.0 years age range = 12–35 years	- Identification of recurring TVC states that partially diverge from static connectivity patterns - Regions belonging to the DMN have highly variable connectivity over time, while regions of the sensory and motor networks exhibit more stable connectivity configurations
Allen et al. (2017)	Siemens Sonata 1.5T Eyes open/Eyes closed 255 volumes TR = 2 s	1. Group ICA decomposition in 43 relevant independent components of interest, classified into seven different functional networks 2. Sliding-window analysis, window length = 30 TRs (60 s), steps = 1 TR (2 s) 3. k-means clustering (5 recurring states) 4. Correlations with EEG data	23 healthy adults 7 females (30.4%) mean age = 29 years SD = 8.8 years	- States were replicable with those of Allen et al. (2014) - TVC states correspond to neurophysiological mental states detected with EEG - Eyes open/eyes closed conditions show some common and some different connectivity patterns Connectivity between the thalamus and the cortex changes from positive to negative in eyes closed vs. open condition
Cabral et al. (2017)	Siemens Avanto 1.5T 180 volumes TR = 2 s	1. Segmentation in 90 cortical brain regions of the AAL atlas 2. Phase-coherence connectivity at each time point 3. Leading eigenvectors and subsequent k-means clustering (five recurring states)	55 healthy adults with good cognitive performance 31 females (44.6%) mean age = 64 years SD = 9 years 43 healthy adults with poor cognitive performance 29 females (66%) mean age = 66 SD = 8 years	- More frequent switches in subjects with poor cognitive vs. good cognitive performances - The lower occurrence of a state of global, positive coherence is associated with worse cognitive performances
Cai B. et al. (2018)	Siemens Trio 3T 126 volumes TR = 3 s	1. Segmentation in 264 regions of the Power atlas (Power et al., 2011), grouped into 10 functional networks 2. Sliding-window analysis, window length = 50 TRs (150 s), steps = 1 TR (3 s) and dynamic sparse connectivity models 3. k-means clustering analysis (4 recurring states)	**Philadelphia neurodevelopmental cohort database** 240 young adults 146 females (60.8%) mean age = 18.99 years SD = 1.12 years 232 children 123 females (53%) mean age = 10.67 years SD = 1.09 years	- Compared with young adults, children had increased connectivity between the DMN and other subnetworks - Children had reduced connectivity among sensorimotor, executive control and auditory networks vs. young adults - Young adults spent more time in the most connected state
Chang and Glover (2010)	GE Signa HDx or Signa 750 3T 360 volumes TR = 2 s	1. ROIs in crucial nodes of the DMN and of the “task-positive” (executive control) network 2. Time-frequency decomposition using Wavelet transform coherence; sliding-window analysis	12 healthy adults 6 females (50%) mean age = 27.7 years SD = 12.4 years	- Coherence and phase between the PCC and nodes of the executive control network significantly vary in time and frequency - High variability over time was observed between the PCC and brain areas involved in higher-level cognitive functions
Chen T. et al. (2016)	Siemens Skyra 3T Eyes open 1,200 volumes TR = 0.72 s Test-retest data	1. Segmentation in 264 regions of the Power atlas (Power et al., 2011) 2. Sliding-window analysis, window length = 55 TRs (40 s), steps = 1 TR (0.72 s) 3. Graph theoretical analysis	**Human Connectome Project dataset** 77 healthy adults 50 females (64.1%) age range = 22–35 years	- The salience network showed highly flexible connectivity with fronto-parietal, cingulate-opercular, and attention networks - The salience network maintained a consistently high level of network centrality over time
Choe et al. (2017)	**Multi-Modal MRI Reproducibility Resource (Kirby) data set** Philips Achieva 3T 210 volumes TR = 2 s Test-retest data **Human Connectome Project S500 Data dataset** Siemens Skyra 3T 1,200 volumes TR = 0.72 s Test-retest data	**Kirby dataset**: 1. Group ICA decomposition in 39 relevant components of interest, classified into 7 functional networks 2. Sliding-window analysis, window length = 30 TRs (60 s) **Human Connectome Project S500 Data dataset**: 1. Group ICA decomposition in 50 relevant components of interest 2. Sliding-window analysis, window lengths = 15, 30, 60, and 120 TRs (11, 22, 43, and 86 s) 3. TVC mean and variance, k-means clustering (three recurring states) and dynamic conditional correlation approaches	**Kirby dataset** 20 healthy adults **Human Connectome Project S500 Data dataset** 523 healthy adults	- TVC can be reliably estimated in test-retest data - The dynamic conditional correlation method seems to be more reliable than sliding-window analysis
Lim et al. (2018)	Siemens Prisma 3T Eyes open 250 volumes TR = 2 s	1. Segmentation of 114 regions of the Yeo atlas (Yeo et al., 2011), classified into 17 functional networks 2. Sliding-window analysis, window length = 7 TRs (14 s), steps = 1 TR (2 s) 3. k-means clustering (3–7 recurring states)	21 healthy adults with high-trait mindfulness 13 females (61.9%) mean age = 23.7 years SD = 3.4 years 18 healthy adults with low-trait mindfulness 13 females (72.2%) mean age = 21.9 years SD = 2.3 years	- High trait mindfulness subjects spent significantly more time in a high within-network connectivity state, characterized by greater anti-correlations between task-positive networks and the DMN - Transitions between brain states was more frequent in high vs. low trait mindfulness subjects
Lindquist et al. (2014)	Philips Achieva 3T 210 volumes TR = 2 s Test-retest data	1. Segmentation of six spherical ROIs (radius = 3 mm) containing regions of the DMN 2. Point-process analysis 3. Estimation of variance of dynamic connectivity correlations, compared with traditional sliding-window analysis	**Multimodal MRI Reproducibility Resource (Kirby21) dataset** 21 healthy adults 10 females (47.6%) mean age = 31.76 years SD = 9.47 years	- Dynamic conditional correlations are able to quantify dynamics of RS fMRI data - Dynamic conditional correlations have a similar performance as sliding-window analysis in quantifying TVC between brain regions
Liu and Duyn (2013)	Multicenter 3T scanners Volumes varying from 119 to 195 TR varying from 2.3 to 3 s	1. Segmentation of two spherical ROIs (radius = 6 mm) containing the PCC and left intraparietal sulcus 2. Point-process analysis 3. k-means clustering of coactivation patterns (eight coactivation patterns for the PCC and 12 for the left intraparietal sulcus)	**1000 Functional Connectomes Project (FCP)** 247 healthy adults 151 females (61.1%) mean age = 22.72 years SD = 4.61 years age range = 18–44 years	- Point-process analysis was able to extract correlational patterns in RS fMRI data from relatively brief periods of co-activation (or co-deactivation) of brain regions - Co-activation patterns resembled classical networks derived from static RS FC analysis, while more fine-grained co-activation patterns were detected
Marusak et al. (2017)	GE Signa 3T Siemens Verio 3T both scanners: 180 volumes TR = 2 s	1. Group ICA decomposition in 25 relevant independent components of interest, classified into 3 functional networks 2. Sliding-window analysis, window length = 22 TRs (44 s), steps = 1 TR (2 s) 3. k-means clustering (six recurring states) 4. Correlation with age and internal thoughts	**Stanford University dataset** 73 normally developing children 34 females (46.57%) mean age = 12.47 SD = 1.88 years **Wayne State University dataset** 73 normally developing children 49 females (67.12%) mean age = 12.09 years SD = 2.54 years	- The occurrence and amount of time spent in specific TVC states are related to the content of self-generated thought during the scan - Temporal variability of TVC among cognitive networks increases with age - Regions showing the highest TVC include multi-modal areas associated with high-order cognitive functions, such as the precuneus and inferior parietal lobe
Marusak et al. (2018)	Siemens Verio 3T 390 volumes TR = 1.5 s	1. Group ICA decomposition in four relevant independent components of interest 2. Sliding-window analysis, window length = 30 TRs (45 s), steps = 1 TR (1.5 s) 3. k-means clustering (5 recurring states) 4. Correlations with mindfulness scores	42 children 23 females (54.8%) mean age = 10.3 years SD = 2.9 years age range = 6–17 years	- High-mindfulness children had a greater number of transitions between states than low-mindfulness children and showed a state-specific reduction in connectivity between salience/emotion and central executive networks
Nini et al. (2017)	Siemens Trio 3T 225 volumes TR = 2.48 s	1. Segmentation in 90 regions of the AAL atlas 2. Sliding-window analysis, window length = 25 s, steps = 0.6 s 3. Graph theory analysis: flexibility and variance	**1,000 Functional Connectomes Project** 148 healthy young adults 74 females (50%) age range = 18–26 years	- Flexibility of amygdala, hippocampus, fusiform gyrus, and temporal gyrus was higher in males than in females - Flexibility of middle cingulate cortex, thalamus, precuneus, and temporo-occipital regions was higher in females than in males
Shi et al. (2018)	Siemens Trio 3T 232 volumes TR = 2 s	1. Group ICA decomposition in 5 relevant independent components of interest 2. Sliding-window analysis, window length = 30 TRs (60 s), steps = 1 TR (2 s) 3. k-means clustering (four recurring states) and fuzzy-meta states analyses	**Southwest University Longitudinal Imaging Multimodal dataset** 331 healthy young adults 247 females (74.6%) mean age = 20.20 years SD = 1.34 years 212 healthy young adults 115 females (54.2%) mean age = 22.36 years SD = 1.49 years	- Subjects having a high score in subjective well being spent less time in a state characterized by low cross-network connectivity and strong within-network connectivity - The total number of transitions across states was correlated with a higher subjective well-being score
Smith et al. (2018)	Siemens Skyra 3T scanner Eyes open 1,200 volumes TR = 0.72 s Test-retest data	1. Segmentation of 90 regions from Shirer et al. (Shirer et al., 2012) 2. Point-process analysis 3. k-means clustering of coactivation patterns (four recurring states)	**Human Connectome Project S500 Data dataset** 100 healthy adults 54 females (54%)	- Brain state- properties were reliable across days - Summary metrics of brain connectivity dynamics had an adequate test-retest reliability
Tagliazucchi et al. (2013)	Siemens Trio 3T 1,505 volumes TR = 2.08 s	1. Group ICA decomposition in six relevant independent components of interest 2. Detrended fluctuation analysis 3. Hurst exponent (measuring long-range temporal dependence)	39 healthy adults	- Temporal memory of RS fMRI time series decreases from wakefulness to deep non-rapid eye movement sleep - Long-range temporal dependence decreases especially in regions of the DMN and attention network
Vidaurre et al. (2018)	**Human Connectome Project dataset** Siemens Skyra 3T Eyes open 1,200 volumes TR = 0.72 s **UK Biobank dataset** Siemens Skyra 3T Eyes open 490 volumes TR = 0.735 s	1. Group ICA decomposition in 50 relevant independent components of interest from the HCP dataset, in 55 relevant independent components of interest from the UK Biobank dataset 2. Hidden Markov model 3. Stochastic inference (12 recurring states)	**Human Connectome Project dataset** 820 healthy adults 453 females (55.2%) age range = 22–35 years **UK Biobank dataset** 5847 healthy adults age range = 40–69 years	- Hidden Markov models allow to model resting (or task-related) brain activity as a time-varying sequence of distinct brain networks, also when analyzing very large amounts of data
Yaesoubi et al. (2015a)	Data from Allen et al., 2014 Siemens Trio 3T 152 volumes TR = 2 s	1. Group ICA decomposition in 50 relevant independent components of interest Time-frequency decomposition 2. k-means clustering (five recurring states)	Data from Allen et al. (2014) 405 healthy adults 3. 200 females (49.4%) 4. mean age = 21.0 years 5. age range = 12–35 years	- A new time-frequency decomposition approach, based on wavelet transform coherence, detected time-frequency connectivity variations in RS fMRI data - Recurring connectivity patterns in time-frequency domain revealed significant between-group differences based on sex
Yaesoubi et al. (2015b)	Data from Allen et al. (2014) Siemens Trio 3T 152 volumes TR = 2 s	1. Group decomposition in 50 relevant components of interest, classified into seven different functional networks 2. Sliding-window analysis, window length = 32 TRs (44 s), steps = 1 TR (2 s) 3. Clustering of sliding-window matrices using temporal ICA to find maximally mutually temporally independent connectivity patterns (five recurring states) 4. Sex differences	Data from Allen et al. (2014) 405 healthy adults 200 females (49.4%) mean age = 21.0 years age range = 12–35 years	- A method alternative to k*-*means clustering is proposed, based on temporal ICA. This method allowed to detect temporally independent connectivity states - Frequency of occupancy of such states was not different between genders
Yaesoubi et al. (2017b)	Data from Allen et al. (2014) Siemens Trio 3T 152 volumes TR = 2 s	1. Group ICA decomposition in 50 relevant independent components of interest 2. Time-frequency decomposition 3. k-means clustering of z-scored time-frequency decompositions to find recurring frequency modes (four recurring modes)	Data from Allen et al. (2014) 405 healthy adults 200 females (49.4%) mean age = 21.0 years age range = 12–35 years	- Time-frequency decomposition allowed to capture frequency variations in individual network time courses - Frequency modes represent “periodic” activities consisting of instantaneous activations and deactivations
Yang et al. (2014)	Siemens Trio 3T 884 volumes TR = 0.645 s Test-retest data	1. Four spherical ROIs (radius = 3 mm) in crucial nodes of the posteromedial cortex; segmentation of 156 regions from Craddock et al. (2012) 2. Sliding-window analysis, window length = 69 TRs (44 s), steps = 3 TRs (2 s) 3. Hierarchical clustering (five recurring states)	22 healthy adults 4. 6 females (27.3%) 5. mean age = 33.5 years 6. SD = 12.5 years 7. age range = 19–60 years	- Each subregion of the posteromedial cortex was associated with five recurring connectivity states Each subregion possessed a unique preferred state and distinct transition patterns
Zalesky et al. (2014)	Siemens Skyra 3T 1,200 volumes TR = 0.72 s	1. Segmentation in different numbers of ROIs (from 90 to 4,000) (Zalesky et al., 2010) 2. Sliding-window analysis, window length = 60 s, steps = 1 TR (0.72 s) 3. Non-stationarity of RS fMRI fluctuations measured using an *ad hoc* test statistic	**Human connectome project Q2 Data dataset** 10 healthy adults 6 females (60%) age range = 22–35 years	- A consistent set of functional connections had pronounced fluctuations over time - The most dynamic connections were inter-modular and involved hubs of the DMN and fronto-parietal network
Zhang C. et al. (2018)	Siemens Skyra 3T 1,200 volumes TR = 0.72 s Test-retest data	1. Segmentation in 116 regions of the AAL atlas and 160 regions of the Dosenbach atlas (Dosenbach et al., 2010) Sliding-window analysis, window length = from 20 TRs to 200 TRs 2. Standard deviation from the mean and excursion from the median. Amplitude of low-frequency fluctuations across sliding windows	**Human connectome project S900 Data dataset** 820 healthy adults 454 females (55.4%) age range = 22–37 years	- TVC was reliable, especially when windows size was between 30 and 50 TRs, but less reliable than static FC - The highest reliability for static and dynamic FC analysis was found for intra-network connections in the fronto-parietal, DMN, sensorimotor, and occipital networks

Ω *All RS scans were acquired in the eyes-closed condition, except where indicated*.

∧ *TVC analysis approach summarizes: (1) ROIs used; (2) assessment of time-varying correlations between brain regions; (3) features extracted for assessing TVC*.

θ *For each study group of healthy subjects, sex is represented as number of females (%), mean age and standard deviation (SD)*.

*RS, resting state; fMRI, functional magnetic resonance imaging; TVC, time-varying functional connectivity; ICA, independent component analysis; TR, repetition time; SD, standard deviation; EEG, electroencephalographic registration; AAL, automated anatomical labeling; ROIs, regions of interest; DMN, default-mode network; PCC, posterior cingulate cortex; HCP, Human connectome project; UK, United Kingdom; FC, functional connectivity*.

In healthy subjects, it was always possible to identify a certain number of recurring connectivity configurations (from 3 to 12, depending on the method applied and on RS fMRI sequence settings). The DMN was one of the functional networks showing the highest degree of connectivity change over time, both when analyzing within-DMN TVC (Chang and Glover, 2010; Liu and Duyn, 2013; Lindquist et al., 2014; Zalesky et al., 2014) and when considering connections between the DMN and other crucial cognitive networks (Chang and Glover, 2010; Liu and Duyn, 2013; Allen et al., 2014; Marusak et al., 2017; Vidaurre et al., 2018). High dynamism was also observed in multimodal brain regions, involved in high-order emotional and cognitive processing (Yang et al., 2014; Zalesky et al., 2014; Chen S. et al., 2016; Marusak et al., 2017; Vidaurre et al., 2018). Such quick temporal reconfigurations may be required to facilitate transient psychological states between different brain functions (starting, maintenance or conclusion of the different attentional, cognitive, and executive tasks). Conversely, networks involved in sensory and motor processing showed more “static” connectivity profiles (Allen et al., 2014; Zalesky et al., 2014).

TVC was also useful to characterize age- and sex-related features. For instance, it was shown that children have higher TVC between the DMN and other subnetworks than young adults, but that young adults have stronger TVC than children among sensorimotor, executive control, and auditory networks (Cai B. et al., 2018). Moreover, variability of TVC among cognitive networks increased with age (Marusak et al., 2017). Overall, these results suggest that maturation is associated with a higher flexibility of functional connections. More discrepancies were found when analyzing sex-related characteristics of connectivity dynamics (Yaesoubi et al., 2015a,b; Nini et al., 2017). While some studies found no differences in TVC configurations between males and females (Yaesoubi et al., 2015b), other studies found that connectivity configurations were different between genders (Yaesoubi et al., 2015a,b; Nini et al., 2017): males showed a higher connectivity flexibility than females in the amygdala, hippocampus, fusiform, and temporal gyrus, whereas the opposite trend was found in the middle cingulate cortex, thalamus, precuneus, and some temporal-occipital regions (Nini et al., 2017).

TVC constitutes a complex and novel methodology. Studies from healthy controls also served to test how reliable and reproducible TVC results were across scanning sessions. This was the goal of some recent investigations (Choe et al., 2017; Smith et al., 2018; Zhang C. et al., 2018), which found that TVC metrics were reliable across days (Smith et al., 2018) and had an overall good reproducibility (Choe et al., 2017; Smith et al., 2018; Zhang C. et al., 2018), even if lower than that of the corresponding static FC metrics (Zhang C. et al., 2018). The highest reliability was found for intra-network connections in the DMN, fronto-parietal, sensorimotor, and occipital networks (Zhang C. et al., 2018).

To better investigate the intrinsic nature of TVC states and their electrophysiological correlates, simultaneously acquired electroencephalography (EEG) and RS fMRI data were analyzed and concurrent temporal variations were assessed (Allen et al., 2017). Results indicated that connectivity states detected by TVC analysis correspond to neuro-electric brain activity with distinct spectral signatures. Moreover, eyes open/eyes closed conditions show some common and some different connectivity patterns, with a greater integration within sensory systems, as well as reduced modularity and increased global efficiency, in the eyes-closed compared to the eyes-open condition (Allen et al., 2017). These results integrate and complete previous EGG/RS fMRI studies, which showed a variable TVC configuration between wakefulness and different stages of sleep (Tagliazucchi et al., 2013), with temporal memory and long-range temporal dependencies decreasing from wakefulness to deep non-rapid eye movement sleep.

### Correlations Between TVC and Behavioral/Neuropsychological Performances in Healthy Subjects

To date, correlations between TVC measures and cognitive performances in healthy controls have been evaluated by one study (Cabral et al., 2017), which found that worse cognitive performance in healthy elderly was associated with a lower permanence in a TVC state characterized by strong, positive connectivity. These results suggest that a more static pattern of TVC may characterize poor vs. good performers.

Another study (Shi et al., 2018) analyzed the correlation between TVC and scores obtained at questionnaires of subjective well-being, and found that subjects with higher well-being scores spent less time in low cross-network and strong within-network connectivity states. The total number of transitions between states was also higher in subjects with high well-being scores, suggesting a more efficient transfer of information between networks in this group (Shi et al., 2018). Finally, two studies assessing the relationship between TVC and mindfulness in healthy adults (Lim et al., 2018) and children (Marusak et al., 2018) had similar conclusions, showing that high-mindfulness subjects spent more time in highly-connected states and switched more frequently between states than low-mindfulness subjects, suggesting a more efficient and flexible connectivity in the first group.

## Application of TVC Techniques to MS

### Main TVC Findings in MS Patients

The main studies assessing TVC abnormalities in MS patients are summarized in Table 2. As it is evident from this table, TVC methodologies applied in MS investigations were quite heterogeneous. Despite this, results of different studies share some common points.

**Table 2:** Summary of studies assessing time-varying resting state functional connectivity modifications in multiple sclerosis (MS).

**Study**	**RS fMRI acquisition parameters^Ω^**	**TVC analysis approach^**∧**^**	**Study subjects^**θ**^**	**Main findings**
Bosma et al. (2018)	GE 3T 277 volumes TR = 2 s	1. Segmentation of 5 cortical regions belonging to the DMN, salience network, ascending and descending nociceptive network (according with Hemington et al., 2016) and the primary sensory area (Harvard Oxford Cortical Structural Atlas, Desikan et al., 2006) 2. Dynamic conditional correlations 3. Standard deviation of dynamic conditional correlation and of RS fMRI time series	31 MS patients (25 relapsing-remitting MS, 4 secondary progressive MS, 3 unknown) 20 females (64.5%) mean age = 39 years SD = 10 years 31 healthy controls 20 females (64.5%) mean age = 38 years SD = 11 years	- Greater TVC between the salience and ascending nociceptive network in MS patients vs. healthy controls - Greater variability of RS FC in MS patients vs. healthy controls - Patients with neuropathic pain had abnormal cross-network connectivity between the salience and DMN
d'Ambrosio et al. (2019)	Multicenter setting: seven centers 3T 200 volumes TR = 3 s	1. Group ICA decomposition in 43 relevant independent components of interest, classified into seven different functional networks 2. Sliding-window analysis, window length = 22 TRs (66 s), step = 1 TR (3 s) 3. k*-*means clustering analysis (three recurring states); fuzzy meta-state analysis 4. Correlations with clinical variables, cognitive performance, T2 lesion volume, and brain volume	**MAGNIMS Cognition study** 62 relapsing-remitting MS patients (23 with cognitive impairment, 39 without cognitive impairment) 40 females (64.5%) mean age = 39.5 years SD = 8.5 years 65 healthy controls 38 females (58%) mean age = 35.8 years SD = 9.4 years	- MS patients, compared to healthy controls, showed: (i) reduced TVC between subcortical and visual/cognitive networks, as well as between visual and cognitive networks; and (ii) increased TVC between subcortical and sensorimotor networks - Compared to cognitively preserved, cognitively impaired MS patients showed reduced TVC between subcortical and DMN, lower dwell time in a state characterized by high intra- and inter-network connectivity, and lower global connectivity variations over time - In patients with cognitive impairment, reduced global dynamism correlated with brain atrophy
Huang et al. (2019)	Siemens Trio 3T 240 volumes TR = 2 s	1. Segmentation of six regions of interest belonging to the attention network 2. Sliding-window analysis, window length = 40 TRs (80 s), and 20 TRs (40 s), steps = 1 TR (2 s) 3. Estimation of the temporal correlation coefficient between truncated time courses	22 relapsing-remitting MS patients 15 females (68.2%) mean age = 39.1 years age range = 20–58 years 22 healthy controls 15 females (68.2%) mean age = 39.6 years age range = 26–56 years	- Compared to controls, decreased TVC within the dorsal and ventral attention networks, as well as increased TVC between the dorsal and ventral attention networks was detected - Decreased TVC within parietal and between fronto-temporal regions was correlated with a higher white matter lesion load
Leonardi et al. (2013)	Siemens Trio 3T 450 volumes TR = 1.1 s	1. Segmentation of 88 brain regions from the AAL atlas (Tzourio-Mazoyer et al., 2002) 2. Sliding-window analysis, window length = 30 TRs (33 s), 40 TRs (44 s), 60 TRs (66 s) and 120 TRs (132 s), steps = 2 TRs (2.2 s) 3. Principal component analysis: 10 eigenconnectivity patterns (states)	22 relapsing-remitting MS patients 14 females (63.6%) mean age = 36.8 years SD = 8 years 14 healthy controls 9 females (64.2%) mean age = 38.4 years SD = 6 years	- A novel data-driven approach, based on principal component analysis, was able to detect large-scale recurring connectivity patterns with similar dynamics - Compared to controls, MS patients showed more frequently strong connections in parietal regions (PCC, superior parietal and angular gyrus) and more frequently weak connections in prefrontal regions and in the amygdala
Lin et al. (2018)	Philips Achieva 3T 240 volumes TR = 2 s	1. Segmentation of 18 cortical regions from the Freesurfer Desikan atlas (Desikan et al., 2006) 2. Sliding-window analysis, window length = 20 TRs (40 s), steps = 1 TR (2 s) 3. Graph theory: network variations, flexibility of inter-hemispheric, cross-hemispheric, and intra-hemispheric connections	37 relapsing-remitting MS patients 28 females (75.7%) mean age = 42.57 years SD = 11.4 years 18 relapsing-remitting MS patients (matched with healthy controls) 15 females (83.3%)mean age=32 years SD=4.9 years 15 healthy controls 8 females (53.3%) mean age = 28.93 years SD = 5 years	- Lower network variations and higher flexibility of inter-hemispheric connections in MS patients compared with controls - Better executive functions on cognitive testing were associated to higher connectivity dynamics
Rocca et al. (2019)	Philips Achieva 1.5T 200 volumes TR = 3 s	1. Group ICA decomposition in 43 relevant independent components of interest, classified into seven different functional networks 2. Sliding-window analysis, window length = 22 TRs (66 s), step = 1 TR (3 s) 3. k-means clustering analysis (two recurring states) 4. Fuzzy meta-state analysis 5. Correlations with clinical variables, cognitive performance, T2 lesion volume, and brain volume	50 patients with CIS suggestive of MS 30 females (60%) mean age = 30.5 years SD = 7.7 years 13 healthy controls 9 females (69.2%) mean age = 33.1 years SD = 7.8 years	- At baseline, compared to healthy controls, CIS patients showed TVC abnormalities between sensorimotor and DMN with the remaining networks - According to type of onset, selective baseline RS FC decrease was detected in functional networks more affected by the clinical attack - At follow-up, increased connectivity strength and global connectivity dynamism was observed in patients vs. healthy controls - In CIS patients, higher TVC at year 2 correlated with lower white matter lesion volume changes at follow-up
van Geest et al. (2018a)	GE Signa HDxt 3T RS fMRI: 202 volumes TR = 2.2 s Task-related (SDMT): 460 volumes TR = 2 s	1. Segmentation of 224 regions from the Brainnettome atlas (Fan et al., 2016), Yeo atlas (Yeo et al., 2011) and from FSL FIRST segmentation 2. RS fMRI: sliding-window analysis, window length = 27 TRs (59.4 s), steps = 5 TRs (11 s) Task-related fMRI: sliding-window analysis, window length = 30 TRs (60 s), steps = 5 TRs (10 s) 3. Sum of the absolute differences in RS and task-related FC between consecutive windows	29 MS patients 18 females (62%) mean age = 41.25 years SD = 9.34 years 18 healthy controls 11 females (61.1%) mean age = 40.68 years SD = 13.29 years	- TVC in the DMN increased during the task vs. rest in both controls and MS patients - A higher increase of TVC in the DMN during the task vs. rest was associated with better information processing speed in MS patients
van Geest et al. (2018b)	Siemens Sonata 1.5T RS fMRI: 200 volumes TR = 2.85 s Task-related fMRI (episodic memory): 208 volumes TR = 2.22 s	1. Segmentation of 92 brain regions from the AAL atlas (Tzourio-Mazoyer et al., 2002) 2. Task-related fMRI: sliding-window analysis, window length = 27 volumes (59.9 s), steps = 5 TRs (11.1 s) 3. Sum of the absolute differences in FC between consecutive windows	38 MS patients 26 females (68.4%) mean age = 47.2 years SD = 8 years 29 healthy controls 18 females (62.1%) mean age = 43.9 years SD = 8.4 years	- TVC of the left and right hippocampus, as well as TVC of the entire brain, did not differ between healthy controls and MS patients - Lower hippocampal TVC was associated with better verbal learning and memory, and with better visuospatial learning and memory performances
Zhou et al. (2016)	Siemens Trio 3T 240 volumes TR = 2 s	1. Voxel-wise analysis (no ROI selection necessary) 2. Calculation of brain entropy and amplitude of low frequency fluctuations 3. Voxel-wise comparison of brain entropy and amplitude of low frequency fluctuations	34 relapsing-remitting MS patients 21 females (61.8%) mean age = 42.1 years age range = 20–58 years 34 healthy controls 21 females (61.8%) mean age = 41.8 years age range = 21–58 years	- Brain entropy was increased in MS patients compared to controls, especially in regions related to motor, executive, spatial coordination and memory functions - More severe brain entropy was correlated with a higher clinical disability

Ω *All RS scans were acquired in the eyes-closed condition*.

∧ *TVC analysis approach summarizes: (1) ROIs used; (2) assessment of time-varying correlations between brain regions; (3) features extracted for assessing TVC*.

θ *For each study group of healthy subjects, sex is represented as number of females (%), mean age and standard deviation (SD)*.

*RS, resting state; fMRI, functional magnetic resonance imaging; TVC, time-varying functional connectivity; TR, repetition time; DMN, default-mode network; MS, multiple sclerosis; SD, standard deviation; AAL, automated anatomical labeling; PCC, posterior cingulate cortex; CIS, clinically isolated syndrome*.

First of all, networks showing the greatest amount of TVC abnormalities in MS patients in comparison to healthy subjects were the DMN, salience, executive and sensorimotor networks (Leonardi et al., 2013; Zhou et al., 2016; Bosma et al., 2018; Lin et al., 2018; d'Ambrosio et al., 2019; Rocca et al., 2019).

The regional pattern of TVC abnormalities was quite complex, and regions involved by TVC changes were variable across studies, probably depending from the used TVC approach and patients' clinical characteristics. The analysis of eigenconnectivity patterns helped to identify the presence of stronger TVC in parietal regions and weaker TVC in frontal/subcortical regions in relapsing-remitting MS patients with mild to moderate disability compared to healthy controls (Leonardi et al., 2013). These patients also showed more frequently strong connections in temporal and parietal (angular gyrus) regions as well as weaker connections in motor and amygdalar regions vs. control subjects (Leonardi et al., 2013). Another study assessing TVC abnormalities in relapsing-remitting MS patients with mild disability found an increased BEN (corresponding to an increased connectivity disorganization) of regions involved in motor, executive and spatial coordination, as well as reduced BEN in memory brain areas (including temporal and hippocampal cortices) and relay areas as the cerebellum or the brainstem compared to healthy subjects (Zhou et al., 2016).

A recent study using DCC to quantify TVC (Bosma et al., 2018) confirmed the results obtained by Leonardi et al. (2013), and found an increased BOLD signal variability in posterior regions of the DMN in MS patients vs. controls. The same study also found an increased TVC between the salience network and the ascending nociceptive pathway. Conversely, divergent results were obtained by Lin et al. (2018), who showed an overall reduction of network variation in MS patients compared to healthy controls, suggesting a globally more “static” FC configuration, but at the same time found an increased flexibility of interhemispheric connections, which was interpreted as a compensatory mechanism for the decreased global connectivity. A complex pattern of increased and decreased TVC was also shown by d'Ambrosio et al. (2019), who found, in a multicenter study, a selective TVC increase between subcortical and visual/cognitive networks, and a TVC decrease between subcortical and sensorimotor networks in relapsing-remitting MS patients compared to healthy controls (d'Ambrosio et al., 2019).

Specific investigations of crucial systems involved in cognitive functions were performed by Van Geest et al., who studied TVC of the hippocampal network (Lin et al., 2018; van Geest et al., 2018a,b) and of the DMN (Lin et al., 2018; van Geest et al., 2018a,b) and by Huang et al., who investigated the attention network (Huang et al., 2019). Overall, hippocampal and DMN TVC were not different between MS patients and control subjects; however, including TVC measures in multivariate statistical models contributed to explain the performance of MS patients at visuospatial memory (Lin et al., 2018; van Geest et al., 2018a,b) and information processing speed (Lin et al., 2018; van Geest et al., 2018a,b) tasks. Huang et al. detected a complex pattern of TVC abnormalities, which was characterized by a TVC decrease within the dorsal and ventral attention networks, as well as TVC increase between the same networks (Huang et al., 2019).

Changes in TVC at the earliest stages of the disease have been rarely assessed, but interesting results have been observed. Patients with clinically isolated syndrome (CIS) suggestive of MS exhibited, early after the first demyelinating attack, reduced TVC in the functional networks more affected by the clinical onset, compared to healthy controls (Rocca et al., 2019). These patients also showed, in the first 2 years after the clinical event, a progressive increase over time of TVC strength, mainly between the DMN and sensorimotor/visual/cognitive networks, combined with a progressive increase over time of global fuzzy meta-state dynamism (Rocca et al., 2019).

Overall, these results suggest that, at the beginning of the disease, TVC dysfunctions have a specific correspondence with clinical symptoms. Then, a progressive increase of TVC oscillations occurs, probably trying to compensate disease-related damage. This initial phase seems to be followed by a loss of coordination and flexibility among brain regions in MS patients (Leonardi et al., 2013; Zhou et al., 2016; Lin et al., 2018; d'Ambrosio et al., 2019), which may be compensated by local increased fluctuations between specific areas (Lin et al., 2018;van Geest et al., 2018a,b).

Recent studies tried to investigate TVC changes in MS populations affected by specific clinical manifestations. In details, cognitive impairment in patients with MS was associated to reduced TVC between subcortical and DMN areas, as well as to reduced global dynamism, compared to cognitively preserved patients (d'Ambrosio et al., 2019). Patients with MS suffering from neuropathic pain expressed selectively reduced TVC strength in the salience-descending nociceptive circuit (Bosma et al., 2018), whereas in patients without such neuropathic pain, TVC strength was increased in the same network (Bosma et al., 2018).

The large majority of the above-mentioned studies assessed TVC changes in relapsing-remitting MS patients, while detailed investigations of TVC abnormalities occurring in progressive MS phenotypes or over the course of the disease, are still missing.

### Correlations Between TVC and Clinical, Neuropsychological, and Structural MRI Variables in MS

Different correlation analyses have been performed in MS patients, in order to understand the possible association between TVC abnormalities and motor and cognitive performances, as well as with specific clinical symptoms such as fatigue.

A higher expanded disability status scale (EDSS) score, reflecting more severe clinical disability, was found to be correlated with increased BEN in the bilateral supplementary motor area and in the right precentral operculum (Zhou et al., 2016), as well as with a more rigid (less fluid) global connectivity in MS patients (Lin et al., 2018). Conversely, other studies failed to show significant associations between TVC abnormalities and disability, probably because of the relatively low sample size and/or a narrow EDSS range (Leonardi et al., 2013).

Several correlations have been detected between TVC abnormalities and MS patients' cognitive performances. In particular, better scores in tests involving executive control functions and processing speed ability were correlated with a higher global network dynamism (Lin et al., 2018). Similar findings were shown by van Geest et al. (2018a), who found that a higher dynamism in the DMN during an information processing speed task vs. a resting state condition was associated with better information processing speed performances. These results are in agreement with the reduced network dynamics observed in cognitively impaired vs. preserved MS patients (d'Ambrosio et al., 2019). Conversely, a lower hippocampal TVC contributed to explain, at least partially, better verbal learning, visuospatial learning, and memory performances (van Geest et al., 2018b).

Lower fatigue was associated with reduced TVC in the parahippocampal gyrus, right posterior cerebellum, and brainstem (Zhou et al., 2016). Pain interference has been associated with increased TVC in the posterior cingulate cortex, an associative region involved in the salience and nociceptive networks and DMN (Bosma et al., 2018).

A few studies investigated the relationship between TVC and white matter lesions or MS-related structural damage. Decreased TVC between parietal and fronto-temporal regions of the attention network was associated with an higher lesion load in relapsing-remitting MS patients (Huang et al., 2019). A significant association has been demonstrated between reduced global dynamism in cognitively impaired MS patients and brain atrophy (d'Ambrosio et al., 2019), as well as between increased TVC and diffuse microstructural damage in relapsing-remitting MS patients, quantified as a higher mean diffusivity on diffusion-tensor MRI (Zhou et al., 2016). At the earliest stages of MS, a progressive increase of TVC over 2 years of follow-up was associated with a lower white matter lesion volume change over the same period of time (Rocca et al., 2019).

In summary, in MS patients, abnormal global TVC properties of the sensorimotor, DMN and salience networks were associated with more severe tissue damage at structural MRI, more severe clinical disability, worse cognitive performance and pain interference, evidencing a maladaptive neuronal response to direct disease-related damage. Conversely, abnormal TVC properties of the temporal network and relay areas as the cerebellum and brainstem were correlated with better cognitive performances and less severe fatigue, suggesting a compensatory role of TVC changes.

## Application of Time-Varying FC Techniques to Psychiatric and Other Neurological Diseases

### Main Findings in Psychiatric and Other Neurological Diseases

The main studies discussed in this section are summarized in Table 3.

**Table 3:** Summary of studies assessing time-varying resting state functional connectivity modifications in different psychiatric and neurological pathologies (excluding multiple sclerosis).

**Study**	**RS fMRI acquisition parameters^Ω^**	**TVC analysis approach^**∧**^**	**Study subjects^**θ**^**	**Main findings**
Abrol et al. (2017)	Six sites: Siemens Tim Trio 3T One site: GE Discovery MR750 3T 162 volumes TR = 2 s	1. Group ICA decomposition in 47 relevant independent components of interest, classified into 7 functional networks 2. Sliding-window analysis, window length = 22 TRs (44 s), steps = 1 TR (2 s) 4. Clustering (five recurring states) performed using temporal ICA 3. Correlations with gray matter volumes	**FBIRN Data Repository** 151 schizophrenia patients 37 females (24.5%) mean age = 37.8 years 163 healthy subjects 46 females (28.2%) mean age = 36.9 years	- Compared to healthy subjects, patients with schizophrenia exhibited higher TVC strength between: i) sensorimotor, precuneus and parietal areas; and ii) frontal, temporal and insular cortices - In patients, TVC abnormalities correlated with lower gray matter volumes
Alderson et al. (2018)	Philips Intera MR 3T 140 volumes TR = 3 s	1. Group ICA decomposition in nine relevant independent components of interest, subsequently segmented in 148 cortical regions from the Destrieux atlas (Destrieux et al., 2010) 2. Time-frequency analysis 3. Graph theory: synchrony, global metastability, eigenvector centrality, clustering coefficient, local efficiency, and participation coefficient 4. Correlation with structural abnormalities	**ADNI database** 34 patients with Alzheimer's disease 18 females (52.9%) mean age = 73.79 years SD = 6.14 years 33 patients with mild cognitive impairment 13 females (39.4%) mean age = 73.61 years SD = 5.6 years 36 healthy controls 19 females (52.8%) mean age = 74.46 years SD = 5.51 years	- In Alzheimer's disease patients, reduced synchrony was observed between right fronto-parietal regions, sensorimotor regions and DMN, together with overall reduced metastability - In patients, increased eigenvector centrality, clustering coefficient, local efficiency, and participation coefficient correlated with more severe structural damage
Cai J. et al. (2018)	Siemens Trio 3T Unspecified volumes TR = 2 s	1. Segmentation of 76 brain regions from the Desikan atlas (Desikan et al., 2006) 2. Sliding-window analysis, window length = 30 TRs (60 s), steps = 2 TRs (4 s) 3. Graph theoretical analysis: global efficiency, clustering efficiency, modularity, assortativity, Fiedler value	69 Parkinson's disease patients 30 females (43.5%) mean age = 60 years SD = 9.8 years 29 healthy controls 13 females (43.5%) mean age = 58.3 years SD = 97.5 years	- Compared to healthy subjects, patients with Parkinson's disease showed lower network connections (Fiedler value), modularity and global efficiency - Lower network connections in patients with Parkinson's disease correlated with disease severity
Cetin et al. (2016)	Siemens Trio 3T 149 volumes TR = 2 s	1. Group ICA decomposition in 39 relevant independent components of interest 2. Sliding-window analysis, window length = 31 TRs (62 s), steps = 1 TRs (2 s) k-means clustering (five recurring states) 3. Correlations with magnetoencephalography data and classification performance compared to static FC and magnetoencephalography	47 schizophrenia patients 13 females (27.7%) mean age = 35.18 years SD = 11.83 years 45 healthy controls 7 females (15.6%) mean age = 37.28 years SD = 13.86 years	- Classification between schizophrenia patients and healthy controls improved with TVC (accuracy = 82.79%) compared to static FC metrics (accuracy = 70.33%) - Classification performance did not improve when using a combination of TVC and magnetoencephalography metrics (accuracy = 85.35%), compared to the combination of static FC and magnetoencephalography metrics (accuracy = 87.91%)
Chen et al. (2018)	Philips Achieva 3T 170 volumes TR = 2 s	1. Segmentation of left and right primary motor area, premotor cortex and supplementary motor area (spherical ROIs, radius = 5 mm) Sliding-window analysis, window length = 32TRs (64 s), steps = 1 TR (2 s) 2. Standard deviation of TVC across windows	70 stroke patients 45 right-sided lesions 23 females (32.9%) mean age = 58.44 years SD = 11.43 years 25 left-sided lesions 8 females (11.4%) mean age = 59.88 years SD = 12.96 years 55 healthy controls 26 females (37.1%) mean age = 56.73 years SD = 10.21 years	- Compared to healthy controls, stroke patients showed TVC reductions between sensorimotor and visual-related cortices and between the sensorimotor and the limbic system - In stroke patients with right-sided lesions, reduced TVC between the right primary motor area and the left precentral gyrus correlated with more severe disability
Damaraju et al. (2014)	6 sites: Siemens Tim Trio 3T 1 site: GE Discovery MR750 3T 162 volumes TR = 2 s	1. Group ICA decomposition in 50 relevant independent components of interest, classified into 7 different functional networks 2. Sliding-window analysis, window length = 22 TRs (44 s), steps = 1 TR (2 s) 3. k-means clustering (five recurring states)	151 schizophrenia patients 37 females (24.5%) mean age = 37.8 years 163 healthy subjects 46 females (28.2%) mean age = 36.9 years	- Compared to healthy controls, schizophrenia patients showed: (i) higher dwell time in states characterized by overall low inter- and intra-network TVC strength; (ii) lower dwell time in states characterized by high correlations between visual, motor and auditory networks; and (iii) increased TVC between thalami and sensory networks
Diez-Cirarda et al. (2018)	Philips Achieva TX 3T 214 volumes TR = 2.1 s	1. Group ICA decomposition in 29 relevant independent components of interest, classified into seven functional networks 2. Sliding-window analysis, window length = 22 TRs (44.2 s), steps = 1 TR (2.1 s) 3. k-means clustering (two recurring states). Graph theory: global efficiency, local efficiency, clustering coefficient, betweenness centrality	37 patients with Parkinson's disease 12 with normal cognition 6 females (50%) mean age = 65.17 years SD = 8.31 years 23 with mild cognitive impairment 10 females (44%) mean age = 69.17 years SD = 4.48 years 26 healthy controls 8 females (31%) mean age = 68.31 years SD = 7.52 years	- Compared to healthy controls, Parkinson's disease patients with mild cognitive impairment showed lower dwell time in a state characterized by overall low strength of inter- and intra-network connections, as well as higher number of transitions between states - Parkinson's disease patients with cognitive impairment also showed: (i) reduced clustering coefficient in the right precentral gyrus vs. healthy controls; and (ii) reduced betweenness centrality of the left paracentral gyrus vs. patients without cognitive impairment
Du et al. (2017)	3 sites: Siemens Trio Tim 3T 2 sites: GE Signa HDx 3T 1 site: Siemens Allegra 3T 1 site: Philips 3T 100–210 volumes TR ranging from 1.5 to 3 s	1. Segmentation of 116 brain regions from the AAL atlas (Tzourio-Mazoyer et al., 2002) 2. Sliding-window analysis, window length = 20 TRs (ranging from 30 to 60 s) 3. GIG-ICA clustering (five recurring-states) 4. Correlations with cognitive scores	**Bipolar and schizophrenia network on intermediate phenotypes** 113 schizophrenia patients 57 females (50%) mean age = 35.57 years SD = 12.29 years 132 schizoaffective disorder patients 75 females (57%) mean age = 36.23 years SD = 12.23 years 140 bipolar disorder with psychosis patients 87 females (62%) mean age = 36 years SD = 12.57 years 238 healthy controls 138 females (58%) mean age = 38.15 years SD = 12.55 years	- Compared to healthy controls (and bipolar patients), schizophrenia and schizoaffective disorder patients showed increased TVC between frontal with angular and postcentral areas, and reduced TVC between temporal and frontal areas - Compared with all remaining study groups, schizophrenia patients also showed reduced TVC between the cerebellum and subcortical and frontal areas - Reduced TVC between cerebellar and frontal areas correlated with higher symptom severity scores
Du et al. (2018)	Siemens Tim Trio 3T 180 volumes TR = 2 s	1. Segmentation of 116 brain regions from the AAL atlas (Tzourio-Mazoyer et al., 2002) 2. Sliding-window analysis, window length = 20 TRs (40 s), steps = 1 TR (2 s) 3. GIG-ICA clustering (five recurring-states)	58 schizophrenia patients 20 females (35%) mean age = 21.8 years SD = 3.8 years 53 adults at high risk of developing schizophrenia 21 females (38%) mean age = 20.4 years SD = 4.5 years 70 healthy controls 29 females (41%) mean age = 21.9 years SD = 5.6 years	- Compared to healthy controls, schizophrenia patients and adults with high risk of developing schizophrenia showed TVC alterations between motor, temporal, cerebellar, frontal and thalamic areas - Schizophrenia patients, compared to adults with high risk of developing schizophrenia, also showed increased TVC between the cerebellum, temporal cortex, frontal gyri and thalami - Increased TVC between temporal and cerebellar areas correlated with higher symptom severity scores
Engels et al. (2018)	GE Signa HDxT 3T 202 volumes TR = 2.15 s	1. Segmentation of 264 brain regions from the Power atlas (Power et al., 2011) 2. Sliding-window analysis, window length = 28 TRs (60.2 s), steps = 5 TRs (10.75 s) 3. Standard deviation of TVC across windows	24 Parkinson's disease patients 7 females (29.2%) mean age = 63.42 years SD = 7.93 years 27 healthy controls 11 females (40.1%) mean age = 59.37 years SD = 8.54 years	- Compared with patients without cognitive impairment, Parkinson's disease patients with mild cognitive impairment showed higher TVC between the DMN and the rest of the brain - In patients, no correlation was found between TVC abnormalities and motor severity
Falahpour et al. (2016)	17 sites TR = 2 s	1. Manual segmentation of 10 spherical ROIs (radius = 6 and 10 mm) 2. Sliding-window analysis, window length = 15 TRs (30 s), steps = 4 TRs (8 s) 3. Standard deviation of TVC across windows	**Autism Brain Imaging Data Exchange (ABIDE)** 76 autism spectrum disorders 9 females (11.8%) mean age = 16.1 years SD = 4.9 years range = 7–29.9 years 76 typically development young adults 12 females (15.8%) mean age = 15.8 years SD = 4.5 years range = 8–29.9 years	- No between-group differences were observed in TVC
Fu et al. (2018)	6 sites: Siemens Tim Trio 3T 1 site: GE Discovery MR750 3T 162 volumes TR = 2 s	1. Group ICA decomposition in 48 relevant independent components of interest, classified into seven functional networks 2. Sliding-window analysis, window length = 20 TRs (40 s), steps = 1 TR (2 s) 3. k-means clustering of dynamic amplitude of low-frequency fluctuations (six recurring states)	**FBIRN Data Repository** 151 schizophrenia patients 37 females (24.5%) mean age = 37.8 years SD = 11.4 years 163 healthy controls 46 females (28.2%) mean age = 36.9 years SD = 11 years	- Compared to healthy controls, schizophrenia patients showed increased dynamic amplitude of low-frequency fluctuations in states characterized by strong TVC between the thalami and sensory regions - Patients also showed reduced dynamic amplitude of low-frequency fluctuations in states characterized by weak TVC between the thalami and sensory regions
Guo et al. (2018)	14 sites	1. Manual segmentation of three spherical ROIs (radius = 6 mm) 2. Flexible least squares to construct a TVC map at each timepoint 3. k-means clustering (five recurring states) Correlations with clinical scores	**Autism Brain Imaging Data Exchange (ABIDE)** 209 autism spectrum disorder adolescents 0 females (0%) mean age = 16.5 years SD = 6.2 years 298 typical development adolescents 0 females (0%) mean age = 16.8 years SD = 6.2 years	- Compared to typically developing adolescents, autism spectrum disorder adolescents showed reduced TVC among the right anterior insula, ventromedial prefrontal cortex and the posterior central cortex - Reduced TVC between the right anterior insula and the ventromedial prefrontal cortex correlated with higher symptom severity
He et al. (2018)	Philips Achieva 3T TR = 2 s	1. Group ICA decomposition of the DMN, used to select the PCC for subsequent analyses 2. Sliding-window analysis, window length = 50 TRs (100 s), steps = 2 TRs (4 s) 3. Calculation of TVC map between the PCC and the rest of the brain in each window; calculation of variance of FC across windows k-means clustering analysis (six recurring states) Correlation with social behavior scales	**Autism Brain Imaging Data Exchange (ABIDE)** 38 autism spectrum disorders 0 females (0%) age range = 3–7 years 41 typical development children 0 females (0%) age range = 3–6 years	- Compared to typically developing children, Autism spectrum disorders children showed differences in TVC variance between the PCC and: (1) the whole brain; (2) the right precentral gyrus; and (3) visual areas - In autism spectrum disorder children, lower TVC variance between the PCC and the right precentral gyrus negatively correlated with social motivation
Jie et al. (2018)	Philips 3T scanners 140 volumes TR ranging from 2.2 to 3.1 s	1. Segmentation of 116 brain regions from the AAL atlas (Tzourio-Mazoyer et al., 2002) 2. Sliding-window analysis, non-overlapping windows, window length ranging from 30 to 60 s 3. Metrics of temporal and spatial variability of TVC across windows 4. Six machine-learning classification algorithms	**ADNI database** 43 patients with mild cognitive impairment with late onset 17 females (39.5%) mean age = 72.1 years SD = 8.2 years 56 patients with mild cognitive impairment with early onset 35 females (62.5%) mean age = 71.1 years SD = 6.8 years 50 healthy controls 29 females (58%) mean age = 75 years SD = 6.9 years	- Patients with early mild cognitive impairment, compared to healthy controls, showed increased TVC variability - TVC abnormalities helped to identify patients with early-onset mild cognitive impairment from patients with late-onset mild cognitive impairment and healthy controls (accuracy = 74.7 and 73.6%, respectively)
Jones et al. (2012)	GE Signa HDx 3T 100 volumes TR = 3 s	1. Group ICA decomposition in 54 relevant independent components of interest, used to develop 68 cubical ROIs (edge = 10 mm) 2. Sliding-window analysis, window length = 9 TRs (27 s) and 11 TRs (33 s) 3. Graph theory: variability of modularity across windows	28 patients with Alzheimer's disease Unspecified sex and age 892 healthy controls 438 females (49%) median years = 79 years range = 75–83 years	- Patients with Alzheimer's disease showed lower dwell time in brain states with strong contributions of the posterior areas of the DMN, and higher dwell time in states with strong contributions of the anterior areas of the DMN
Klugah-Brown et al. (2018)	GE Discovery MR750 3T 250 volumes TR = 2 s	1. Group ICA decomposition in 50 relevant independent components of interest, classified into seven functional networks 2. Sliding-window analysis, window length = 22 TRs (44 s), steps = 1 TR (2 s) 3. k-means clustering (four recurring states) Individual reconstruction of TVC states using dual regression	19 frontal lobe epilepsy patients 9 females (47.4%) median age = 24.2 years range = 13–51 years 18 healthy controls 5 females (27.8%) median age = 23.9 years range = 11–41 years	- Compared to healthy subjects, epilepsy patients showed reduced TVC between the fronto-parietal network and cerebellar/subcortical networks - They also spent less time in the most fundamental connectivity state - A lower dwell time in this state correlated with age of seizure onset
Li et al. (2018)	GE Discovery 750 3T 240 volumes TR = 2 s	1. Segmentation of cortical brain regions from the AAL atlas (Tzourio-Mazoyer et al., 2002) 2. Sliding-window analysis, window length = 50 TRs (100 s), steps = 10 TRs (20 s) 3. Standard deviation of TVC density (proportional to the number of functional connections) across windows	43 children with benign epilepsy (centrotemporal spikes) 19 females (44.2%) mean age = 9.61 years SD = 2.04 years 28 typically developing children 13 females (46.4%) mean age = 10 years SD = 2.31 years	- Compared to typically developing children, epilepsy children showed decreased TVC variability in the orbital inferior frontal gyrus and increased TVC variability in the precuneus - Patients with interictal epileptiform discharges, compared to patients without interictal epileptiform discharges, showed higher TVC variability in the supramarginal gyrus - Excessive TVC variability of the precuneus correlated with a younger onset age of seizure
Liao et al. (2018)	Unspecified GE 3T 240 volumes TR = 2 s	1. Segmentation of 200 brain regions using the Craddock atlas (Craddock et al., 2012) 2. Sliding-window analysis, window length = 50TRs (100 s), steps = 5TRs (10 s) 3. Graph theory analyses: network strength, network efficiency, nodal efficiency, small worldness Variance of area-under-the-curve of graph metrics 4. Correlation with suicide ideation scores	48 major depressive disorder 37 females (77.1%) mean age = 34.8 years SD = 10.3 years 30 healthy controls 18 females (60%) mean age = 35.7 years SD = 10.2 years	- Increased network strength and efficiency in patients with suicide ideation compared to healthy subjects and major depressed patients without suicide ideation - Patients without suicide ideation showed TVC alterations within the left middle/inferior frontal gyrus, right superior parietal gyrus, right postcentral gyrus and right fusiform gyrus - TVC network strength distinguished patients with and without suicide ideation from healthy subjects
Liu et al. (2017)	Siemens Trio 3T 250 volumes TR = 2 s	1. Group ICA decomposition in 21 relevant independent components of interest 2. Sliding-window analysis, window length = 55 TRs (110 s), steps = 1 TR (2 s) 3. k-means clustering (six recurring states) 4. Correlations with disease duration	43 patients with idiopathic generalized epilepsy 15 females (34.8%) mean age = 23.12 years SD = 4.8 years 48 healthy controls 19 females (39.5%) mean age = 23.02 years SD = 1.49 years	- Patients with idiopathic generalized epilepsy showed reduced dwell time in a state characterized by strong correlations between visual and remaining sense-related networks, as well as increased dwell time in a state characterized by strong correlations between cognitive and sense-related networks - In patients with idiopathic generalized epilepsy, reduced dwell time in the first above-mentioned state was correlated with a higher seizure frequency
Liu et al. (2018)	Siemens Trio 3T Unspecified volumes TR = 2 s	1. Segmentation of bilateral putamen and 56 brain regions from the Desikan atlas (Desikan et al., 2006) 2. Sliding-window analysis, window length = 30 TRs (60 s), steps = 2 TRs (4 s) 3. Standard deviation of TVC strength 4. Correlations with clinical scores	30 patients with Parkinson's disease 11 females (36.7%) mean age = 57.8 years SD = 9.9 years 28 healthy controls 14 females (50%) mean age = 58.4 SD = 7.6 years	- Compared to healthy controls, Parkinson's disease patients showed reduced TVC between the posterior subunit in the left putamen with the left superior frontal gyrus, right putamen and the right precentral gyrus, as well as between the right posterior putamen and bilateral pallidum nuclei - TVC abnormalities correlated with more severe disability
Mennigen et al. (2018)	Siemens Trio 3T 170 volumes TR = 2 s	1. Group ICA decomposition in 47 relevant independent components of interest, classified into eight functional networks 2. Sliding-window analysis, window length = 22 TRs (44 s), steps = 1 TR (2 s) 3. k-means clustering (five recurring states). Fuzzy meta-state analysis	53 patients with clinical high-risk for psychosis 21 females (39.6%) mean age = 20.4 years SD = 4.5 years 58 schizophrenia patients 20 females (34.5%) mean age = 21.8 years SD = 3.8 years 70 healthy controls 41 females (58.6%) mean age = 21.9 years SD = 5.6 years	- Compared to healthy subjects, schizophrenia patients showed significantly lower global meta-state dynamism - Compared to healthy controls, patients with high-risk for psychosis showed significantly lower meta-state dynamism
Qiu et al. (2018)	GE Excite 3T 195 volumes TR = 2 s	1. Segmentation of three amygdalar subregions in each hemisphere, following the JuBrain Cytoarchitectonic Atlas (Zilles and Amunts, 2010) 2. Sliding-window analysis, window length = 100 TRs (200 s), step = 1 TR (2 s) 3. Voxel-wise maps of variance of amygdalar TVC across windows	30 patients with major depression disorder 20 females (66.7%) mean age = 36.1 years SD = 12.3 years range = 18–60 years 62 healthy controls 33 females (53.2%) mean age = 35.1 years - SD = 15.9 years - range = 16–81 years	- Compared to healthy controls, patients with major depression disorder exhibited decreased positive TVC correlations between the amygdala and left centromedial and superficial subregions, primarily in the brainstem, decreased positive fronto-thalamic TVC, and decreased negative TVC of the left centromedial subregion with the right superior frontal gyrus - In patients, mean positive TVC strength between the left centromedial region and brainstem was positively correlated with the age of onset of major depression disorder
Quevenco et al. (2017)	Philips Achieva 7T 200 volumes TR = 2 s	1. Segmentation of 90 brain cortical regions from the AAL atlas (Tzourio-Mazoyer et al., 2002) 2. Sliding-window analysis, window length = 30 TRs (60 s), steps = 1 TR (2 s) 3. Principal components analysis: eigen-connectivity patterns (states)	37 healthy controls divided according to presence/absence of memory decline 13 females (35.1%) mean age = 73 years SD = 6.6 years	- Subjects with memory decline showed reduced TVC between anterior and posterior brain areas - Increased global connectivity, reduced TVC between anterior and posterior brain areas, increased TVC between interhemispheric fronto-temporal areas and reduced TVC between parietal and temporal areas correlated with memory decline and apoprotein E-ε4 carrier status
Rashid et al. (2014)	Siemens Allegra 3T Eyes open 202 volumes TR = 1.5 s	1. Group ICA decomposition in 49 relevant independent components of interest, classified into 7 functional networks 2. Sliding-window analysis, window length = 22 TRs (33 s), steps = 1 TR (1.5 s) 3. k-means clustering (five recurring states)	60 schizophrenia patients 13 females (21.7%) mean age = 35.85 years SD = 12.01 years 38 bipolar disorder patients 20 females (52.6%) mean age = 38.96 years SD = 10.9 years 61 healthy controls 28 females (45.9%) mean age = 35.4 years SD = 11.57 years	- Compared to controls, schizophrenia patients showed increased TVC between: (i) temporal regions; (ii) frontal regions; (iii) subcortical regions; iv) temporal and parietal regions, and reduced TVC between: (i) frontal and parietal regions and (ii) frontal and occipital areas - Compared to bipolar patients, schizophrenia patients showed increased TVC between: (i) frontal and parietal areas; (ii) sensorimotor areas; (iii) sensorimotor and parietal areas - Compared to healthy controls, bipolar disorder patients showed increased TVC between temporal and parietal areas, as well as reduced TVC within parietal regions
Rashid et al. (2016)	Siemens Allegra 3T Eyes open 202 volumes TR = 1.5 s	1. Group ICA decomposition in 49 relevant independent components of interest, classified into seven functional networks 2. Sliding-window analysis, window length = 22 TRs (33 s), steps = 1 TR (1.5 s) 3. k-means clustering (five recurring-states) 4. Machine learning classification of the study subgroups	60 schizophrenia patients 13 females (21.7%) mean age = 35.85 years SD = 12.01 years 38 bipolar disorder patients 20 females (52.6%) mean age = 38.96 years SD = 10.9 years 61 healthy controls 28 females (45.9%) mean age = 35.4 years SD = 11.57 years	- TVC improved classification between patients with schizophrenia, patients with bipolar disorder and healthy controls: TVC overall classification accuracy (84.28%) was significantly higher than overall classification accuracy of static FC metrics (59.12%)
Rashid et al. (2018a)	GE Discovery 3T 160 volumes TR = 2 s	1. Group ICA decomposition in 38 relevant independent components of interest 2. Sliding-window analysis, window length = 22 TRs (44 s), steps = 1 TR (2 s) 3. k-means clustering (four recurring states) 4. Sex and age association with recurring-states	**Generation R study** 774 children 22 children diagnosed with autism spectrum disorders 15 children with autistic traits age range = 4.89–8.90 years 774 typical development children	- In typically developing children, TVC globally increased with age in fronto-temporal, fronto-parietal and temporo-parietal networks - Compared to typically developing children, autism spectrum disorder children showed: (i) increased TVC between the right insula and left superior frontal gyrus, right supramarginal gyrus and left precuneus; and (ii) reduced TVC between the right insula and the right supramarginal gyrus, the left supplementary motor area and right supramarginal gyrus - Autism spectrum disorder patients with high level of autistic traits showed longer dwell times in a globally disconnected state
Rashid et al. (2018b)	Siemens Trio 3T GE Discovery MR750 3T 162 volumes TR = 2 s	1. Group ICA decomposition in 7 relevant independent components of interest 2. Sliding-window analysis, window length = 22 TRs (44 s), steps = 1 TR (2 s) 3. k-means clustering (five recurring states) Correlation with peak weights of single nucleotide polymorphism mostly located in chromosome 6	**FBIRN Data Repository** 61 schizophrenia patients 9 females (14.8%) mean age = 38.4 years 87 healthy controls 26 females (29.9%) mean age = 36.8 years	- Schizophrenia patients showed a lower occupancy rate of a state characterized by high TVC in temporal, parietal, limbic and occipital regions (state 1), as well as a higher occupancy rate of a state characterized by increased fronto-limbic and intra-occipital TVC (state 5) vs. healthy subjects - Schizophrenia patients with increased gene polymorphism had stronger disrupted TVC in states 1 and 5
Ridley et al. (2017)	Siemens Avanto 1.5T 200 volumes TR = 3 s	1. Segmentation of spherical ROIs (radius = 5 mm), defined by their contact to implanted electrodes 2. Sliding-window analysis, window length = 30 TRs (90 s), steps = 0.66 TR (2 s) 3. Correlation with EEG data	9 patients with drug-resistant epilepsy 3 females (33.3%) mean age = 30.4 years SD = 4.5 years range = 24–38 years No control group	- In cortices not involved by epilepsy, TVC was correlated with EEG registration of all frequency bands - In epileptic cortices, TVC correlated with EEG in alpha band
Sakoglu et al. (2010)	Siemens Allegra 3T Active fMRI (auditory oddball task): Two consecutive runs 249 volumes TR = 1.5 s	1. Group ICA decomposition in 10 relevant independent components of interest 2. Sliding-window analysis, window length = 64 TRs (96 s), steps = 2 TRs (3 s) 3. Time-frequency analysis 4. Standard deviation of TVC across windows, between-group comparison of TVC in each window	28 schizophrenia patients 5 females (17.9%) mean age = 36.4 years SD = 12.43 years 28 healthy controls 9 females (32.1%) mean age = 28.8 years SD = 10.7 years	- Compared to controls, schizophrenia patients exhibited reduced TVC task-modulation between the medial temporal network and the right lateral fronto-parietal/frontal networks. They also showed increased TVC task-modulation between the motor and frontal networks, and between the posterior DMN and orbitofrontal/parietal networks
Sun et al. (2018)	**Laboratory dataset** Philips Achieva 3T Eyes open 240 volumes TR = 2 s **COBRE dataset** Siemens Trio 3T 150 volumes TR = 2 s	1. Segmentation of 90 brain regions from the AAL atlas (Tzourio-Mazoyer et al., 2002) 2. Sliding-window analysis, window length = 50 TRs (100 s), steps = 3 TRs (6 s) 3. Graph theory analysis: temporal global/local efficiency, richness, sparsity range of temporal networks	**Laboratory dataset** 18 schizophrenia patients 8 females (44.4%) mean age = 38.8 years SD = 9.9 years range = 24–56 years 19 healthy controls 9 females (47.4%) mean age = 37.7 years SD = 9.0 years range = 28–59 years **COBRE dataset** 53 schizophrenia patients 12 females (22.6%) mean age = 38.3 years SD = 13.9 years range = 18–65 years 57 healthy controls 20 females (35.1%) mean age = 35.4 years SD = 11.9 years range = 18–62 years	- Compared to healthy controls, schizophrenia patients showed higher temporal regional efficiency with left frontal, right medial parietal and bilateral subcortical areas - Abnormalities of temporal network efficiency correlated with a higher presence of schizophrenia positive and negative symptoms
Vergara et al. (2018)	Siemens Trio 3T 145 volumes TR = 2 s	1. Group ICA decomposition in 48 relevant independent components of interest, classified in nine functional networks 2. Sliding-window analysis, window length = 15 TRs (30 s) 3. k-means clustering (four recurring states) 4. Machine learning for group classification	48 patients with mild traumatic brain injury 25 females (52.1%) mean age = 27.79 years SD = 9.18 years 48 healthy controls 25 females (52.1%) mean age = 27.40 years SD = 8.96 years	- Compared to healthy controls, mild traumatic brain injury patients showed stronger TVC between the cerebellum and sensorimotor areas, as well as a trend toward increased connectivity between the cerebellum and almost all cortical areas - Results were similar to those obtained with the study of static FC (Vergara et al., 2017)
Wang et al. (2018)	Siemens TIM Trio 3T 300 volumes TR = 2 s	1. Voxel-by-voxel calculation of connection strength index (CSI) and connection count index (CCI) within a whole gray matter from the MNI template (Evans et al., 1992) 2. Sliding-window analysis, window length = 60 TRs (120 s), steps = 1 TR (2 s) 3. Mean of CSI and CCI across windows	18 patients with juvenile myoclonic epilepsy 15 females (83.3%) mean age = 30.11 years SD = 7.73 years range = 20-48 years 25 young adults 10 females (40%) mean age = 33.2 years SD = 13.5 years	- Patients with juvenile myoclonic epilepsy showed increased TVC in the left dorsolateral prefrontal cortex, dorsal striatum, precentral and middle temporal gyri
Yaesoubi et al. (2017a)	Siemens Tim Trio 3T GE Discovery MR750 3T 162 volumes TR = 2 s	1. Group ICA decomposition in 50 relevant independent components of interest, using data from a subgroup of 120 healthy subjects 2. Time-frequency analysis 3. k-means clustering (five recurring states)	**FBIRN Data Repository** 163 healthy subjects 46 females (28.2%) mean age = 36.9 years 151 schizophrenia patients 37 females (24.5%) mean age = 37.8 years	- Using temporal and frequency information, it was possible to estimate TVC states present both in healthy controls and schizophrenia patients (characterized by very high or very low frequency profiles), and states present just in one group - Compared to controls, schizophrenia patients showed more connectivity patterns characterized by anti-correlations between the sensorimotor and visual/auditory/subcortical networks, as well as more lagged correlation between the DMN and sensory networks
Yu et al. (2015)	Siemens Trio 3T Eyes open 150 volumes TR = 2 s	1. Group ICA decomposition in 48 relevant independent components of interest, classified into six functional networks 2. Sliding-window analysis, window length = 20 TRs (40 s), step = 1 TR (2 s) 3. Graph theory: connectivity strength, clustering coefficient, global efficiency; variance of graph metrics over time. 4. Assessment of reoccurring connectivity states based on graph metrics (four recurring states)	82 schizophrenia patients 17 females (20.7%) mean age = 38 years SD = 14 years 82 healthy controls 19 females (23.2%) mean age = 37.7 years SD = 10.8 years	- Compared to controls, schizophrenia patients showed lower connectivity strength, clustering coefficient and global efficiency, as well as higher occupancy rate of a state characterized by disconnection between the sensorimotor, the cognitive control, and the DMN
Yue et al. (2018)	Siemens Trio 3T 240 volumes TR = 2 s	1. Segmentation of bilateral amygdalae, using stereotaxic and probabilistic maps of cytoarchitectonic boundaries 2. Sliding-window analysis, window length = 18 TRs (36 s) 3.Standard deviation of voxel-wise amygdalar TVC across windows	33 schizophrenia patients 22 females (66.7%) mean age = 30.6 years SD = 8.13 years 34 healthy controls 20 females (58.8%) mean age = 28.12 years SD = 6.5 years	- Compared to controls, schizophrenia patients showed increased TVC between the left amygdala and orbitofrontal regions - In schizophrenia patients, variability of TVC correlated with worse information processing and attention performance, as well as with more severe disease severity
Zhang W. et al. (2018)	Siemens Trio 3T 1,000 volumes TR = 0.427 s	1. Segmentation of Brodmann areas 44, 45 (frontal), 22, 40 (auditory) (Zilles and Amunts, 2010) 2. Sliding-window analysis, window length 100 TRs (42.7 s), steps = 2 TRs (0.85 s) 3. k-means clustering (5 recurring states) 4. Variance of TVC strength between ROIs across windows. 5. Correlation with clinical scales	35 schizophrenia patients 14 females (40%) mean age = 32.61 years SD = 11.58 years 22 healthy controls 13 females (60%) mean age = 34.91 years SD = 13.34 years	- Schizophrenia patients with auditory hallucinations showed decreased TVC between the left frontal speech and left temporal auditory areas vs. healthy controls
Zhi et al. (2018)	**Multicenter setting** Philips Achieva 3T Siemens Verio 3T Siemens Prisma 3T 240 volumes TR = 2 s	1. Group ICA decomposition in 49 relevant independent components of interest, classified into eight functional networks 2. Sliding-window analysis, window length = 22 TRs (44 s), steps = 1 TR (2 s) 3. k-means clustering (five recurring states) 4. Graph theory: global and node properties in each connectivity state 5. Correlations with depression severity and cognitive score	182 major depressive disorder patients 119 females (65.4%) mean age = 32.0 years SD = 10.3 years 218 healthy controls 142 females (65.2%) mean age = 29.5 years SD = 8.3 years	- Compared to controls, major depressive disorder patients showed: (i) higher TVC strength between the superior frontal and middle frontal gyrus; (ii) decreased TVC between the lingual gyrus and middle occipital gyrus; and (iii) decreased TVC between the superior parietal lobe and middle frontal gyrus - Correlation between TVC abnormalities and: (i) more severe depressive symptoms, impaired attention and worse executive functions; (ii) lower attention; and (iii) worse performances at working memory and executive functions

Ω *All RS scans were acquired in the eyes-closed condition, except where indicated*.

∧ *TVC analysis approach summarizes: (1) ROIs used; (2) assessment of time-varying correlations between brain regions; (3) features extracted for assessing TVC*.

θ *For each study group of healthy subjects, sex is represented as number of females (%), mean age and standard deviation (SD)*.

*RS, resting state; fMRI, functional magnetic resonance imaging; TVC, time-varying functional connectivity; TR, repetition time; ICA, independent component analysis; FBIRN, function biomedical informatics research network data; ADNI, Alzheimer's disease neuroimaging initiative; SD, standard deviation; DMN, default-mode network; FC, functional connectivity; ROI, region of interest; GIG-ICA, group-information-guided ICA; AAL, automated anatomical labeling; COBRE, center for biomedical research excellence; EEG, electroencephalographic registration; PCC, posterior cingulate cortex; CSI, connection strength index; CCI, connection count index; MNI, Montreal Neurological Institute*.

Several studies tried to characterize TVC abnormalities present in different psychiatric and neurological diseases, sometimes looking for an early diagnostic biomarker (Du et al., 2018; Mennigen et al., 2018). Modification of TVC strength, dwell time or number of transitions between states varied according to the disease status in patients affected by bipolar disorder (Rashid et al., 2014, 2016), schizophrenia (Yu et al., 2015; Cetin et al., 2016; Rashid et al., 2016; Gazula et al., 2018; Yue et al., 2018; Zhang W. et al., 2018), depression (Liao et al., 2018; Qiu et al., 2018; Zhi et al., 2018), autism (He et al., 2018; Rashid et al., 2018a), stroke (Chen et al., 2018), mild traumatic brain injury (Vergara et al., 2018), epilepsy (Ridley et al., 2017; Klugah-Brown et al., 2018), Alzheimer's disease (Quevenco et al., 2017; Jie et al., 2018), and Parkinson's disease (Engels et al., 2018).

In psychiatric diseases, widespread TVC abnormalities have been found. Patients with bipolar disorder and major depression expressed TVC abnormalities mainly in executive (Rashid et al., 2014; Du et al., 2017), amygdala/salience (Qiu et al., 2018; Zhi et al., 2018), and salience/executive regions (Mokhtari et al., 2018a,b). Schizophrenia patients showed a complex pattern of decreased and increased TVC mainly in the DMN (Sakoglu et al., 2010; Abrol et al., 2017), and in frontal, parietal, auditory (Damaraju et al., 2014; Rashid et al., 2014; Du et al., 2017, 2018; Sun et al., 2018), visual (Fu et al., 2018; Rashid et al., 2018b; Sun et al., 2018), and thalamic areas (Damaraju et al., 2014; Rashid et al., 2014, 2018b; Du et al., 2018). Schizophrenia patients spent less time and made fewer transitions between states characterized by weak correlations between the thalami and sense-related brain regions (Damaraju et al., 2014). They also showed more lagged correlations between the DMN and sensory networks (Yaesoubi et al., 2017a) and a higher occupancy rate of globally disconnected states (Yu et al., 2015; Cetin et al., 2016; Rashid et al., 2016; Gazula et al., 2018; Yue et al., 2018; Zhang W. et al., 2018). In children with autism spectrum disorders, TVC was mainly decreased in DMN and insular areas (Falahpour et al., 2016; Guo et al., 2018; He et al., 2018; Rashid et al., 2018a).

In neurological disorders, TVC abnormalities have been mainly observed in areas directly affected by the disease. For example, subcortical stroke and mild traumatic brain injury patients showed TVC abnormalities in sensorimotor networks (Chen et al., 2018; Vergara et al., 2018). Patients with myoclonic/frontal lobe epilepsy showed reduced TVC mainly in frontal and parietal brain regions, whereas patients with temporal lobe epilepsy experienced TVC decrease mainly in temporal regions (Ridley et al., 2017; Klugah-Brown et al., 2018; Wang et al., 2018). Generalized epilepsy was related to TVC strength changes mainly in the DMN and cognitive networks (Liu et al., 2017; Li et al., 2018). Patients suffering from Alzheimer's disease had reduced regional (nodal) TVC (Alderson et al., 2018) and alterations in inter-network TVC of the anterior and posterior regions of the DMN (Jones et al., 2012; Quevenco et al., 2017), the frontal cortex and temporal areas (Jie et al., 2018). Patients with Parkinson's disease showed TVC changes mainly in sensorimotor, executive, cognitive (Liu et al., 2018), visual, and DMN areas (Diez-Cirarda et al., 2018), combined with reduced global and nodal TVC (Cai J. et al., 2018; Diez-Cirarda et al., 2018).

### Correlations Between TVC and Clinical, Neuropsychological, and Structural MRI Variables in Psychiatric and Other Neurological Diseases

In schizophrenia patients, reduced global time-resolved graph metrics have been related to structural disease-related damage (Yu et al., 2015), while abnormalities in TVC of auditory brain regions have been correlated with the presence of auditory hallucinations (Sun et al., 2018). Hallucinations were also correlated with a more rigid, reduced global dynamism (Miller et al., 2016; Mennigen et al., 2018). Autistic behavior and diagnosis were associated with longer dwell times in a globally disconnected state (Rashid et al., 2018a).

In patients with temporal lobe epilepsy, recurring states characterized by high inter-network TVC expressed reduced dwell time and correlated with an early seizure onset (Klugah-Brown et al., 2018). Interestingly, reduced TVC in the ictal irritative zone was associated to an intracranial EEG connectivity increase in the same epileptic region in alpha, beta and gamma bands (Ridley et al., 2017). In patients with Alzheimer's disease, TVC abnormalities between the anterior and the posterior DMN areas correlated with poorer episodic memory performance (Quevenco et al., 2017), while reductions in global TVC were associated with microstructural tissue damage (Alderson et al., 2018). In patients with Parkinson's disease, TVC abnormalities in the DMN have been associated with memory performance (Engels et al., 2018), while TVC alterations in the putamen were associated with clinical disability (Liu et al., 2018).

At this moment, TVC approaches are applicable only at a group level. However, some preliminary investigations have successfully used TVC abnormalities to classify schizophrenia patients from bipolar patients and/or healthy controls (Cetin et al., 2016; Rashid et al., 2016), suggesting a future application of TVC at an individual level.

## Current Limitations and Future Directions

The field of TVC is relatively new: all main technical developments have been achieved in the last 9 years. Nonetheless, in such short period of time TVC has provided greater insights into fundamental properties of functional networks, and has improved knowledge of the pathophysiological brain reorganization occurring in MS and other neurological and psychiatric diseases.

However, TVC methodology presents some inherent limitations that are likely to be overcome in the next future. Further investigations are also needed to better understand the physiological meaning of TVC fluctuations and their electrophysiological correlates.

### How Reliably Can Time-Varying Fluctuations Be Detected From fMRI Data?

One of the main pitfalls of current TVC analysis approaches consists in the fact that the mere presence of signal fluctuations in an fMRI time series is often taken as an evidence of TVC (Hindriks et al., 2016). This might be not necessarily true: FC values fluctuating over time might be observed just because of noise, or statistical uncertainty. Several measures have been employed to test the effective presence of FC variability in fMRI time series, including variance (Sakoglu et al., 2010), standard deviation (Chang and Glover, 2010), kurtosis (Laumann et al., 2017), or more complex, non-linear measures (Zalesky et al., 2014). Usually, these metrics are compared between real fMRI data and simulated data, constructed *ad-hoc* to have a static FC. If the test is significant, the null hypothesis of stationarity can be rejected, and TVC can be considered to be effectively present in the data.

Results of studies assessing evidence of TVC in RS fMRI data were quite disappointing, showing that the power of TVC detection in typical 10-min RS fMRI acquisitions was relatively low (Leonardi and Van De Ville, 2015; Hindriks et al., 2016; Zhang C. et al., 2018). Solutions to improve the likelihood of detecting TVC might be the choice of appropriate lengths for sliding windows (Leonardi and Van De Ville, 2015) or the concatenation of more RS fMRI sessions (Hindriks et al., 2016). On the other hand, it is possible that measures used to test the hypothesis of dynamism so far might be not fully appropriate (Miller et al., 2018). Indeed, novel wavelet-based metrics (Miller et al., 2018) seem to be more sensitive to capture non-stationarities present in real RS fMRI data.

### Improving Temporal Resolution of fMRI Acquisitions

Results of TVC also depend upon the temporal resolution used to acquire fMRI data. TVC studies usually investigate modifications in RS FC occurring within seconds, by using fMRI volumes acquired with TRs ranging from 1 s to 3 s (Chang and Glover, 2010; Allen et al., 2017; Cabral et al., 2017; Nini et al., 2017; Yaesoubi et al., 2017b; Marusak et al., 2018). Investigations performed on RS fMRI data acquired with a higher sampling rate, e.g., thanks to the use of simultaneous multi-slice imaging techniques, may be more powerful in detecting changing connectivity reconfigurations over time (Choe et al., 2017). Also, the use of ultra-fast fMRI acquisition techniques, such as inverse imaging (Lin et al., 2006), generalized inverse imaging (Boyacioglu and Barth, 2013), or multi-slab echo-volumar imaging (Posse et al., 2013), might constitute an important improvement for TVC. Ultra-fast fMRI allows to acquire a single functional volume covering the whole brain in <300 ms, resembling the results of magnetoencephalography studies (Asslander et al., 2013). Therefore, fMRI scans acquired with ultra-fast techniques do not include physiological aliasing and allow the detection of more accurate BOLD signal responses to neural activity. Seminal studies already showed that ultra-fast fMRI significantly enhanced the sensitivity of mapping RS FC dynamics (Posse et al., 2013).

### Improving TVC Pre- and Post-processing

Regardless of the analysis method, the signal-to-noise ratio of the BOLD signal in RS fMRI is low, especially in small temporal segments (Handwerker et al., 2012). Non-neural processes contaminating RS fMRI time series can affect TVC estimates (Hutchison et al., 2013; Murphy et al., 2013; Preti and Van De Ville, 2017). These confounds often include the effects of motion, cardiac and respiratory activity, and fluctuations in arterial CO_2_ concentration (Hutchison et al., 2013; Murphy et al., 2013; Nikolaou et al., 2016; Glomb et al., 2018). Global signal regression (GSR) may be useful to better denoise RS fMRI time series (Murphy and Fox, 2017); however, it was shown to slightly reduce reliability of the estimated TVC connectivity states (Smith et al., 2018). Moreover, the impact of GSR was spatially heterogeneous across brain regions and was dependent from the amount of global signal magnitude across windows (Xu et al., 2018). As such, caution is suggested in applying GSR to sliding-window correlation analyses, and a control of subjects' mental fluctuations during RS fMRI scanning is recommended (Xu et al., 2018). By applying accurate pre-processing steps on the fMRI data, the rate of artifacts present in the TVC analyses will be minimized (Murphy et al., 2013), thus increasing the quality of the observed findings.

Improvements can still be done not only to pre-processing of RS fMRI time series, but also to TVC post-processing, e.g., by implementing new, accurate methods to estimate changing connectivity over time. Recent papers proposed new approaches to analyse TVC, which aim at capturing change points of connectivity in functional correlation matrices (Cribben et al., 2012; Jeong et al., 2016; Kundu et al., 2018). Other studies introduced tensor-based multilayer community detection algorithms, which are able to describe how organization of functional networks evolves over time (Al-Sharoa et al., 2019). All these methods might be useful to complement TVC information obtained by using more standard, state-of-art methods, such as sliding-window analysis. Finally, improvements can still be done in statistical thresholding strategies. TVC assessment relies on the use of a massive amount of pairwise correlations, stored in series of connectivity matrices, and the best way to perform a proper adjustment for multiple comparisons is still an open issue. Traditional methods of correction for multiple comparisons (Friston et al., 1994) may be too conservative and may suppress all significant results; therefore, different approaches of adjustment for multiple comparisons might be more suitable. For instance, network-based statistic (NBS, Zalesky et al., 2010) was proposed as an alternative method of multiple comparison correction in studies using graph theory, which suffers of similar drawbacks as TVC. NBS has been rarely applied in TVC studies (Diez-Cirarda et al., 2018), probably because the process to construct “components” is not straightforward when connectivity matrices change over time. Future studies investigating new strategies of adjustment for multiple comparisons may propose new solutions for this issue.

### Functional Interpretation of TVC Findings

Although some studies have tried to provide a functional interpretation of TVC output, several questions remain to be answered by future work. Preliminary data found some degree of correspondence between EEG rhythms and TVC frequency content (Allen et al., 2017) and hypothesized that some of the TVC states observed in healthy subjects, especially at the end of RS fMRI sessions, might be related to drowsiness or light sleep (Allen et al., 2014, 2017). A preliminary study assessing contemporary TVC and EEG registrations confirmed the presence of connectivity changes over different phases of sleep, with long-range temporal dependencies becoming weaker during deep sleep (Tagliazucchi et al., 2013). Still, it is not clear why larger TVC oscillations have been registered in functional networks at the beginning of RS fMRI sessions (Allen et al., 2014, 2017). Theories hypothesizing the brain functional “anticipation” (e.g., brain predisposition to switch quickly between different psychological states; Zalesky et al., 2014) might partially explain why more “specialized” functional networks (sensorimotor, auditory, visual) express more constant TVC behavior, while more complex, multi-modal networks express more dynamism. On the other hand, constant TVC oscillations in sensorimotor, auditory and visual networks might only reflect their lower activity during RS (Syed et al., 2017).

From this perspective, additional multi-modal studies integrating information from imaging and electrophysiological modalities are necessary for a better comprehension of the neural origin, mechanisms and function of temporal FC variations, as well as of the physiological meaning of TVC states.

## Conclusions

The analysis of time-varying FC has contributed to provide significant information on intrinsic brain functional organization, both in healthy and diseased conditions, which complements data produced by static FC approaches. TVC seems to be an intrinsic property of the brain with a neural origin, although some open questions still remain about the correct interpretation of TVC output.

In MS patients, TVC helped to better understand the pathophysiological functional reorganization occurring in the brain, with a peculiar involvement of the DMN, salience, sensorimotor, and fronto-temporal networks. TVC abnormalities were partially correlated with more severe tissue damage and more severe clinical disability, while more extensive correlations were found with abnormal cognitive performances. In patients with neurodegenerative and psychiatric conditions, TVC abnormalities of the DMN, attention and executive networks were also associated to stronger clinical manifestations. Overall, these results suggest a maladaptive neuronal response to disease-related damage.

There are still several unmet needs in neurological and psychiatric conditions that TVC analysis may help to address. First, TVC may be useful to identify multi-modal regions, crucial for functional network plasticity, which may constitute possible targets for motor and cognitive neurorehabilitation protocols, as well as for symptomatic or disease-modifying treatments. Second, trajectories of TVC changes over time during the disease course need to be better defined, both in MS and in psychiatric/other neurodegenerative disorders. This may be the topic of future longitudinal studies, or of cross-sectional studies enrolling patients at different disease phases. Finally, it is still unclear whether TVC abnormalities may have a prognostic value on future disease course. The collection of clinical data at medium- or long-term follow-up may allow to define whether some TVC abnormalities are associated with a more favorable/worse disease prognosis.

## Author Contributions

MH wrote the first draft of the manuscript. PV, MF, and MR drafted/revised all sections of the manuscript. MR contributed to the study concept and acted as study supervisor. All authors contributed intellectually to manuscript revision, read, and approved the submitted version.

### Conflict of Interest Statement

PV received speakers' honoraria from ExceMed. MF is Editor-in-Chief of the Journal of Neurology; received compensation for consulting services and/or speaking activities from Biogen Idec, Merck-Serono, Novartis, Teva Pharmaceutical Industries; and receives research support from Biogen Idec, Merck-Serono, Novartis, Teva Pharmaceutical Industries, Roche, Italian Ministry of Health, Fondazione Italiana Sclerosi Multipla, and ARiSLA (Fondazione Italiana di Ricerca per la SLA). MR received speakers honoraria from Biogen Idec, Novartis, Genzyme, Sanofi-Aventis, Teva, Merck Serono, and Roche and receives research support from the Italian Ministry of Health and Fondazione Italiana Sclerosi Multipla. The remaining author declares that the research was conducted in the absence of any commercial or financial relationships that could be construed as a potential conflict of interest.

## References

[B1] Abrol A. Rashid B. Rachakonda S. Damaraju E. Calhoun V. D. (2017). Schizophrenia shows disrupted links between brain volume and dynamic functional connectivity. Front. Neurosci. 11:624. 10.3389/fnins.2017.00624 29163021 PMC5682010

[B2] Alderson T. H. Bokde A. L. W. Kelso J. A. S. Maguire L. Coyle D. (2018). Metastable neural dynamics in Alzheimer's disease are disrupted by lesions to the structural connectome. Neuroimage 183, 438–455. 10.1016/j.neuroimage.2018.08.033 30130642 PMC6374703

[B3] Allen E. A. Damaraju E. Eichele T. Wu L. Calhoun V. D. (2017). EEG signatures of dynamic functional network connectivity states. Brain Topogr. 31, 101–116. 10.1007/s10548-017-0546-2 28229308 PMC5568463

[B4] Allen E. A. Damaraju E. Plis S. M. Erhardt E. B. Eichele T. Calhoun V. D. (2014). Tracking whole-brain connectivity dynamics in the resting state. Cereb. Cortex 24, 663–676. 10.1093/cercor/bhs352 23146964 PMC3920766

[B5] Al-Sharoa E. Al-Khassaweneh M. Aviyente S. (2019). Tensor based temporal and multi-layer community detection for studying brain dynamics during resting state fMRI. IEEE Trans. Biomed. Eng. 66, 659–709. 10.1109/TBME.2018.2854676 29993516

[B6] Asslander J. Zahneisen B. Hugger T. Reisert M. Lee H. L. LeVan P.. (2013). Single shot whole brain imaging using spherical stack of spirals trajectories. Neuroimage 73, 59–70. 10.1016/j.neuroimage.2013.01.065 23384526

[B7] Baker J. T. Dillon D. G. Patrick L. M. Roffman J. L. Brady R. O. Jr. Pizzagalli D. A.. (2019). Functional connectomics of affective and psychotic pathology. Proc. Natl. Acad. Sci. U.S.A. 116, 9050–9059. 10.1073/pnas.1820780116 30988201 PMC6500110

[B8] Bisecco A. Nardo F. D. Docimo R. Caiazzo G. d'Ambrosio A. Bonavita S.. (2018). Fatigue in multiple sclerosis: the contribution of resting-state functional connectivity reorganization. Mult. Scler. 24, 1696–1705. 10.1177/1352458517730932 28911257

[B9] Biswal B. B. Mennes M. Zuo X. N. Gohel S. Kelly C. Smith S. M.. (2010). Toward discovery science of human brain function. Proc. Natl. Acad. Sci. U.S.A. 107, 4734–4739. 10.1073/pnas.0911855107 20176931 PMC2842060

[B10] Bonavita S. Gallo A. Sacco R. Corte M. D. Bisecco A. Docimo R.. (2011). Distributed changes in default-mode resting-state connectivity in multiple sclerosis. Mult. Scler. 17, 411–422. 10.1177/1352458510394609 21239414

[B11] Bosma R. L. Kim J. A. Cheng J. C. Rogachov A. Hemington K. S. Osborne N. R.. (2018). Dynamic pain connectome functional connectivity and oscillations reflect multiple sclerosis pain. Pain 159, 2267–2276. 10.1097/j.pain.0000000000001332 29994989

[B12] Boyacioglu R. Barth M. (2013). Generalized INverse imaging (GIN): ultrafast fMRI with physiological noise correction. Magn. Reson. Med. 70, 962–971. 10.1002/mrm.24528 23097342

[B13] Busatto G. F. (2013). Structural and functional neuroimaging studies in major depressive disorder with psychotic features: a critical review. Schizophr. Bull. 39, 776–786. 10.1093/schbul/sbt054 23615813 PMC3686460

[B14] Cabral J. Vidaurre D. Marques P. Magalhaes R. Silva Moreira P. Miguel Soares J.. (2017). Cognitive performance in healthy older adults relates to spontaneous switching between states of functional connectivity during rest. Sci. Rep. 7:5135. 10.1038/s41598-017-05425-7 28698644 PMC5506029

[B15] Cai B. Zille P. Stephen J. M. Wilson T. W. Calhoun V. D. Wang Y. P. (2018). Estimation of dynamic sparse connectivity patterns from resting state fMRI. IEEE Trans. Med. Imaging 37, 1224–1234. 10.1109/TMI.2017.2786553 29727285 PMC7640371

[B16] Cai J. Liu A. Mi T. Garg S. Trappe W. McKeown M. J.. (2018). Dynamic graph theoretical analysis of functional connectivity in parkinson's disease: the importance of fiedler value. IEEE J. Biomed. Health Inform. 10.1109/JBHI.2018.2875456. [Epub ahead of print]. 30307882

[B17] Calhoun V. D. Miller R. Pearlson G. Adali T. (2014). The chronnectome: time-varying connectivity networks as the next frontier in fMRI data discovery. Neuron 84, 262–274. 10.1016/j.neuron.2014.10.015 25374354 PMC4372723

[B18] Castellazzi G. Debernard L. Melzer T. R. Dalrymple-Alford J. C. D'Angelo E. Miller D. H.. (2018). Functional connectivity alterations reveal complex mechanisms based on clinical and radiological status in mild relapsing remitting multiple sclerosis. Front. Neurol. 9:690. 10.3389/fneur.2018.00690 30177910 PMC6109785

[B19] Cetin M. S. Houck J. M. Rashid B. Agacoglu O. Stephen J. M. Sui J.. (2016). Multimodal classification of schizophrenia patients with MEG and fMRI data using static and dynamic connectivity measures. Front. Neurosci. 10:466. 10.3389/fnins.2016.00466 27807403 PMC5070283

[B20] Chang C. Glover G. H. (2010). Time-frequency dynamics of resting-state brain connectivity measured with fMRI. Neuroimage 50, 81–98. 10.1016/j.neuroimage.2009.12.011 20006716 PMC2827259

[B21] Chen J. Sun D. Shi Y. Jin W. Wang Y. Xi Q.. (2018). Alterations of static functional connectivity and dynamic functional connectivity in motor execution regions after stroke. Neurosci. Lett. 686, 112–121. 10.1016/j.neulet.2018.09.008 30195973

[B22] Chen S. Langley J. Chen X. Hu X. (2016). Spatiotemporal modeling of brain dynamics using resting-state functional magnetic resonance imaging with gaussian hidden markov model. Brain Connect. 6, 326–334. 10.1089/brain.2015.0398 27008543

[B23] Chen T. Cai W. Ryali S. Supekar K. Menon V. (2016). Distinct global brain dynamics and spatiotemporal organization of the salience network. PLoS Biol. 14:e1002469. 10.1371/journal.pbio.1002469 27270215 PMC4896426

[B24] Choe A. S. Nebel M. B. Barber A. D. Cohen J. R. Xu Y. Pekar J. J.. (2017). Comparing test-retest reliability of dynamic functional connectivity methods. Neuroimage 158, 155–175. 10.1016/j.neuroimage.2017.07.005 28687517 PMC5614828

[B25] Craddock R. C. James G. A. Holtzheimer P. E. 3rd Hu X. P. Mayberg H. S. (2012). A whole brain fMRI atlas generated via spatially constrained spectral clustering. Hum. Brain Mapp. 33, 1914–1928. 10.1002/hbm.21333 21769991 PMC3838923

[B26] Cribben I. Haraldsdottir R. Atlas L. Y. Wager T. D. Lindquist M. A. (2012). Dynamic connectivity regression: determining state-related changes in brain connectivity. Neuroimage 61, 907–920. 10.1016/j.neuroimage.2012.03.070 22484408 PMC4074207

[B27] Damaraju E. Allen E. A. Belger A. Ford J. M. McEwen S. Mathalon D. H.. (2014). Dynamic functional connectivity analysis reveals transient states of dysconnectivity in schizophrenia. Neuroimage Clin. 5, 298–308. 10.1016/j.nicl.2014.07.003 25161896 PMC4141977

[B28] d'Ambrosio A. Valsasina P. Gallo A. De Stefano N. Pareto D. Barkhof F.. (2019). Reduced dynamics of functional connectivity and cognitive impairment in multiple sclerosis. Mult. Scler. 10.1177/1352458519837707. [Epub ahead of print]. 30887862

[B29] Deco G. Kringelbach M. L. (2016). Metastability and coherence: extending the communication through coherence hypothesis using a whole-brain computational perspective. Trends Neurosci. 39, 125–135. 10.1016/j.tins.2016.01.001 26833259

[B30] Desikan R. S. Segonne F. Fischl B. Quinn B. T. Dickerson B. C. Blacker D.. (2006). An automated labeling system for subdividing the human cerebral cortex on MRI scans into gyral based regions of interest. Neuroimage 31, 968–980. 10.1016/j.neuroimage.2006.01.021 16530430

[B31] Destrieux C. Fischl B. Dale A. Halgren E. (2010). Automatic parcellation of human cortical gyri and sulci using standard anatomical nomenclature. Neuroimage 53, 1–15. 10.1016/j.neuroimage.2010.06.010 20547229 PMC2937159

[B32] Diez-Cirarda M. Strafella A. P. Kim J. Pena J. Ojeda N. Cabrera-Zubizarreta A.. (2018). Dynamic functional connectivity in Parkinson's disease patients with mild cognitive impairment and normal cognition. Neuroimage Clin. 17, 847–855. 10.1016/j.nicl.2017.12.013 29527489 PMC5842729

[B33] Dosenbach N. U. Nardos B. Cohen A. L. Fair D. A. Power J. D. Church J. A.. (2010). Prediction of individual brain maturity using fMRI. Science 329, 1358–1361. 10.1126/science.1194144 20829489 PMC3135376

[B34] Du Y. Fryer S. L. Fu Z. Lin D. Sui J. Chen J.. (2018). Dynamic functional connectivity impairments in early schizophrenia and clinical high-risk for psychosis. Neuroimage 180(Pt B), 632–645. 10.1016/j.neuroimage.2017.10.022 29038030 PMC5899692

[B35] Du Y. Pearlson G. D. Lin D. Sui J. Chen J. Salman M.. (2017). Identifying dynamic functional connectivity biomarkers using GIG-ICA: application to schizophrenia, schizoaffective disorder, and psychotic bipolar disorder. Hum. Brain Mapp. 38, 2683–2708. 10.1002/hbm.23553 28294459 PMC5399898

[B36] Engels G. Vlaar A. McCoy B. Scherder E. Douw L. (2018). Dynamic functional connectivity and symptoms of parkinson's disease: a resting-state fMRI study. Front. Aging Neurosci. 10:388. 10.3389/fnagi.2018.00388 30532703 PMC6266764

[B37] Engle R. (2002). Dynamic conditional correlation: a simple class of multivariate generalized autoregressive conditional heteroskedasticity models. J. Bus. Econ. Stat. 20, 339–350. 10.1198/073500102288618487

[B38] Evans A. C. Collins D. L. Milner B. (1992). An MRI-based stereotactic atlas from 250 young normal subjects. Soc. Neurosci. 18:408.

[B39] Falahpour M. Thompson W. K. Abbott A. E. Jahedi A. Mulvey M. E. Datko M. (2016). Underconnected, but not broken? Dynamic functional connectivity MRI shows underconnectivity in autism is linked to increased intra-individual variability across time. Brain Connect. 6, 403–414. 10.1089/brain.2015.0389 26973154 PMC4913487

[B40] Fan L. Chu C. Li H. Chen L. Xie S. Zhang Y.. (2016). The human brainnetome atlas: a new brain atlas based on connectional architecture. Cereb. Cortex 26, 3508–3526. 10.1093/cercor/bhw157 27230218 PMC4961028

[B41] Filippi M. Agosta F. Spinelli E. G. Rocca M. A. (2013a). Imaging resting state brain function in multiple sclerosis. J. Neurol. 260, 1709–1713. 10.1007/s00415-012-6695-z 23052604

[B42] Filippi M. Bar-Or A. Piehl F. Preziosa P. Solari A. Vukusic S. (2018). Multiple sclerosis. Nat. Rev. Dis. Primers 4:43 10.1038/s41572-018-0050-3 30410033

[B43] Filippi M. Preziosa P. Rocca M. A. (2017). Brain mapping in multiple sclerosis: Lessons learned about the human brain. Neuroimage 190, 32–45. 10.1016/j.neuroimage.2017.09.021 28917696

[B44] Filippi M. Valsasina P. Misci P. Falini A. Comi G. Rocca M. A. (2013b). The organization of intrinsic brain activity differs between genders: a resting-state fMRI study in a large cohort of young healthy subjects. Hum. Brain Mapp. 34, 1330–1343. 10.1002/hbm.21514 22359372 PMC6870508

[B45] Friston K. J. Worsley K. J. Frackowiak R. S. Mazziotta J. C. Evans A. C. (1994). Assessing the significance of focal activations using their spatial extent. Hum. Brain Mapp. 1, 210–220. 10.1002/hbm.460010306 24578041

[B46] Fu Z. Tu Y. Di X. Du Y. Pearlson G. D. Turner J. A.. (2018). Characterizing dynamic amplitude of low-frequency fluctuation and its relationship with dynamic functional connectivity: an application to schizophrenia. Neuroimage 180(Pt B), 619–631. 10.1016/j.neuroimage.2017.09.035 28939432 PMC5860934

[B47] Fukushima M. Sporns O. (2018). Comparison of fluctuations in global network topology of modeled and empirical brain functional connectivity. PLoS Comput. Biol. 14:e1006497. 10.1371/journal.pcbi.1006497 30252835 PMC6173440

[B48] Gazula H. Baker B. T. Damaraju E. Plis S. M. Panta S. R. Silva R. F.. (2018). Decentralized analysis of brain imaging data: voxel-based morphometry and dynamic functional network connectivity. Front. Neuroinform. 12:55. 10.3389/fninf.2018.00055 30210327 PMC6119966

[B49] Glomb K. Ponce-Alvarez A. Gilson M. Ritter P. Deco G. (2018). Stereotypical modulations in dynamic functional connectivity explained by changes in BOLD variance. Neuroimage 171, 40–54. 10.1016/j.neuroimage.2017.12.074 29294385

[B50] Guo X. Duan X. Suckling J. Chen H. Liao W. Cui Q.. (2018). Partially impaired functional connectivity states between right anterior insula and default mode network in autism spectrum disorder. Hum. Brain Mapp. 40, 1264–1275. 10.1002/hbm.24447 30367744 PMC6865537

[B51] Handwerker D. A. Roopchansingh V. Gonzalez-Castillo J. Bandettini P. A. (2012). Periodic changes in fMRI connectivity. Neuroimage 63, 1712–1719. 10.1016/j.neuroimage.2012.06.078 22796990 PMC4180175

[B52] He C. Chen Y. Jian T. Chen H. Guo X. Wang J.. (2018). Dynamic functional connectivity analysis reveals decreased variability of the default-mode network in developing autistic brain. Autism Res. 11, 1479–1493. 10.1002/aur.2020 30270547

[B53] Hemington K. S. Wu Q. Kucyi A. Inman R. D. Davis K. D. (2016). Abnormal cross-network functional connectivity in chronic pain and its association with clinical symptoms. Brain Struct. Funct. 221, 4203–4219. 10.1007/s00429-015-1161-1 26669874

[B54] Hindriks R. Adhikari M. H. Murayama Y. Ganzetti M. Mantini D. Logothetis N. K.. (2016). Can sliding-window correlations reveal dynamic functional connectivity in resting-state fMRI? Neuroimage 127, 242–256. 10.1016/j.neuroimage.2015.11.055 26631813 PMC4758830

[B55] Huang M. Zhou F. Wu L. Wang B. Guo L. Zhao Y.. (2019). White matter lesion loads associated with dynamic functional connectivity within attention network in patients with relapsing-remitting multiple sclerosis. J. Clin. Neurosci. 65, 59–65. 10.1016/j.jocn.2019.03.034 30940453

[B56] Hutchison R. M. Womelsdorf T. Allen E. A. Bandettini P. A. Calhoun V. D. Corbetta M.. (2013). Dynamic functional connectivity: promise, issues, and interpretations. Neuroimage 80, 360–378. 10.1016/j.neuroimage.2013.05.079 23707587 PMC3807588

[B57] Jeong S. O. Pae C. Park H. J. (2016). Connectivity-based change point detection for large-size functional networks. Neuroimage 143, 353–363. 10.1016/j.neuroimage.2016.09.019 27622394

[B58] Jie B. Liu M. Shen D. (2018). Integration of temporal and spatial properties of dynamic connectivity networks for automatic diagnosis of brain disease. Med. Image Anal. 47, 81–94. 10.1016/j.media.2018.03.013 29702414 PMC5986611

[B59] Jones D. T. Vemuri P. Murphy M. C. Gunter J. L. Senjem M. L. Machulda M. M. (2012). Non-stationarity in the “resting brain's” modular architecture. PLoS ONE 7:e39731 10.1371/journal.pone.0039731 22761880 PMC3386248

[B60] Kelly C. Biswal B. B. Craddock R. C. Castellanos F. X. Milham M. P. (2012). Characterizing variation in the functional connectome: promise and pitfalls. Trends Cogn. Sci. 16, 181–188. 10.1016/j.tics.2012.02.001 22341211 PMC3882689

[B61] Khambhati A. N. Sizemore A. E. Betzel R. F. Bassett D. S. (2018). Modeling and interpreting mesoscale network dynamics. Neuroimage 180(Pt B), 337–349. 10.1016/j.neuroimage.2017.06.029 28645844 PMC5738302

[B62] Klugah-Brown B. Luo C. He H. Jiang S. Armah G. K. Wu Y.. (2018). Altered dynamic functional network connectivity in frontal lobe epilepsy. Brain Topogr. 32, 394–404. 10.1007/s10548-018-0678-z 30255350

[B63] Kundu S. Ming J. Pierce J. McDowell J. Guo Y. (2018). Estimating dynamic brain functional networks using multi-subject fMRI data. Neuroimage 183, 635–649. 10.1016/j.neuroimage.2018.07.045 30048750 PMC6197899

[B64] Laumann T. O. Snyder A. Z. Mitra A. Gordon E. M. Gratton C. Adeyemo B.. (2017). On the stability of BOLD fMRI correlations. Cereb. Cortex 27, 4719–4732. 10.1093/cercor/bhw265 27591147 PMC6248456

[B65] Leonardi N. Richiardi J. Gschwind M. Simioni S. Annoni J. M. Schluep M.. (2013). Principal components of functional connectivity: a new approach to study dynamic brain connectivity during rest. Neuroimage 83, 937–950. 10.1016/j.neuroimage.2013.07.019 23872496

[B66] Leonardi N. Van De Ville D. (2015). On spurious and real fluctuations of dynamic functional connectivity during rest. Neuroimage 104, 430–436. 10.1016/j.neuroimage.2014.09.007 25234118

[B67] Li R. Wang L. Chen H. Guo X. Liao W. Tang Y. L.. (2018). Abnormal dynamics of functional connectivity density in children with benign epilepsy with centrotemporal spikes. Brain Imaging Behav. 10.1007/s11682-018-9914-0. [Epub ahead of print]. 29956102

[B68] Liao W. Li J. Duan X. Cui Q. Chen H. Chen H. (2018). Static and dynamic connectomics differentiate between depressed patients with and without suicidal ideation. Hum. Brain Mapp. 39, 4105–4118. 10.1002/hbm.24235 29962025 PMC6866497

[B69] Lim J. Teng J. Patanaik A. Tandi J. Massar S. A. A. (2018). Dynamic functional connectivity markers of objective trait mindfulness. Neuroimage 176, 193–202. 10.1016/j.neuroimage.2018.04.056 29709625

[B70] Lin F. H. Wald L. L. Ahlfors S. P. Hamalainen M. S. Kwong K. K. Belliveau J. W. (2006). Dynamic magnetic resonance inverse imaging of human brain function. Magn. Reson. Med. 56, 787–802. 10.1002/mrm.20997 16964616

[B71] Lin S. J. Vavasour I. Kosaka B. Li D. K. B. Traboulsee A. MacKay A.. (2018). Education, and the balance between dynamic and stationary functional connectivity jointly support executive functions in relapsing-remitting multiple sclerosis. Hum. Brain Map. 39, 5039–5049. 10.1002/hbm.24343 30240533 PMC6866468

[B72] Lindquist M. A. Xu Y. Nebel M. B. Caffo B. S. (2014). Evaluating dynamic bivariate correlations in resting-state fMRI: a comparison study and a new approach. Neuroimage 101, 531–546. 10.1016/j.neuroimage.2014.06.052 24993894 PMC4165690

[B73] Liu A. Lin S. J. Mi T. Chen X. Chan P. Wang Z. J.. (2018). Decreased subregional specificity of the putamen in Parkinson's Disease revealed by dynamic connectivity-derived parcellation. Neuroimage Clin. 20, 1163–1175. 10.1016/j.nicl.2018.10.022 30388599 PMC6214880

[B74] Liu F. Wang Y. Li M. Wang W. Li R. Zhang Z.. (2017). Dynamic functional network connectivity in idiopathic generalized epilepsy with generalized tonic-clonic seizure. Hum. Brain Mapp. 38, 957–973. 10.1002/hbm.23430 27726245 PMC6866949

[B75] Liu X. Duyn J. H. (2013). Time-varying functional network information extracted from brief instances of spontaneous brain activity. Proc. Natl. Acad. Sci. U.S.A. 110, 4392–4397. 10.1073/pnas.1216856110 23440216 PMC3600481

[B76] Liu Y. Liang P. Duan Y. Jia X. Yu C. Zhang M.. (2011). Brain plasticity in relapsing-remitting multiple sclerosis: evidence from resting-state fMRI. J. Neurol. Sci. 304, 127–131. 10.1016/j.jns.2011.01.023 21349545

[B77] Loitfelder M. Fazekas F. Petrovic K. Fuchs S. Ropele S. Wallner-Blazek M.. (2011). Reorganization in cognitive networks with progression of multiple sclerosis: insights from fMRI. Neurology 76, 526–533. 10.1212/WNL.0b013e31820b75cf 21300967

[B78] Lowe M. J. Beall E. B. Sakaie K. E. Koenig K. A. Stone L. Marrie R. A.. (2008). Resting state sensorimotor functional connectivity in multiple sclerosis inversely correlates with transcallosal motor pathway transverse diffusivity. Hum. Brain Mapp. 29, 818–827. 10.1002/hbm.20576 18438889 PMC6871176

[B79] Lowe M. J. Phillips M. D. Lurito J. T. Mattson D. Dzemidzic M. Mathews V. P. (2002). Multiple sclerosis: low-frequency temporal blood oxygen level-dependent fluctuations indicate reduced functional connectivity initial results. Radiology 224, 184–192. 10.1148/radiol.2241011005 12091681

[B80] Mak L. E. Minuzzi L. MacQueen G. Hall G. Kennedy S. H. Milev R. (2017). The default mode network in healthy individuals: a systematic review and meta-analysis. Brain Connect. 7, 25–33. 10.1089/brain.2016.0438 27917679

[B81] Marusak H. A. Calhoun V. D. Brown S. Crespo L. M. Sala-Hamrick K. Gotlib I. H.. (2017). Dynamic functional connectivity of neurocognitive networks in children. Hum. Brain Mapp. 38, 97–108. 10.1002/hbm.23346 27534733 PMC5796541

[B82] Marusak H. A. Elrahal F. Peters C. A. Kundu P. Lombardo M. V. Calhoun V. D.. (2018). Mindfulness and dynamic functional neural connectivity in children and adolescents. Behav. Brain Res. 336, 211–218. 10.1016/j.bbr.2017.09.010 28887198 PMC5610942

[B83] Mennigen E. Miller R. L. Rashid B. Fryer S. L. Loewy R. L. Stuart B. K.. (2018). Reduced higher-dimensional resting state fMRI dynamism in clinical high-risk individuals for schizophrenia identified by meta-state analysis. Schizophr. Res. 201, 217–223. 10.1016/j.schres.2018.06.007 29907493 PMC6252113

[B84] Miller R. L. Abrol A. Adali T. Levin-Schwarz Y. Calhoun V. D. (2018). Resting-state fMRI dynamics and null models: perspectives, sampling variability, and simulations. Front. Neurosci. 12:551. 10.3389/fnins.2018.00551 30237758 PMC6135983

[B85] Miller R. L. Yaesoubi M. Turner J. A. Mathalon D. Preda A. Pearlson G.. (2016). Higher Dimensional meta-state analysis reveals reduced resting fMRI connectivity dynamism in schizophrenia patients. PLoS ONE 11:e0149849. 10.1371/journal.pone.0149849 26981625 PMC4794213

[B86] Mokhtari F. Laurienti P. J. Rejeski W. J. Ballard G. (2018a). Dynamic fMRI connectivity tensor decomposition: a new approach to analyze and interpret dynamic brain connectivity. Brain Connect. 9, 95–112. 10.1089/brain.2018.0605 30318906 PMC6390668

[B87] Mokhtari F. Rejeski W. J. Zhu Y. Wu G. Simpson S. L. Burdette J. H.. (2018b). Dynamic fMRI networks predict success in a behavioral weight loss program among older adults. Neuroimage 173, 421–433. 10.1016/j.neuroimage.2018.02.025 29471100 PMC5911254

[B88] Murphy K. Birn R. M. Bandettini P. A. (2013). Resting-state fMRI confounds and cleanup. Neuroimage 80, 349–359. 10.1016/j.neuroimage.2013.04.001 23571418 PMC3720818

[B89] Murphy K. Fox M. D. (2017). Towards a consensus regarding global signal regression for resting state functional connectivity MRI. Neuroimage 154, 169–173. 10.1016/j.neuroimage.2016.11.052 27888059 PMC5489207

[B90] Nikolaou F. Orphanidou C. Papakyriakou P. Murphy K. Wise R. G. Mitsis G. D. (2016). Spontaneous physiological variability modulates dynamic functional connectivity in resting-state functional magnetic resonance imaging. Philos. Trans. Ser. A Math. Phys. Eng. Sci. 374:20150183. 10.1098/rsta.2015.0183 27044987

[B91] Nini M. Hongna Z. Zhiying L. Li Y. Xia W. (2017). Gender differences in dynamic functional connectivity based on resting-state fMRI, in Conference proceedings: Annual International Conference of the IEEE Engineering in Medicine and Biology Society (Stougthon, WI: The Printing House, Inc.) 2017, 2940–2943. 10.1109/EMBC.2017.8037473 29060514

[B92] Posse S. Ackley E. Mutihac R. Zhang T. Hummatov R. Akhtari M.. (2013). High-speed real-time resting-state FMRI using multi-slab echo-volumar imaging. Front. Hum. Neurosci. 7:479. 10.3389/fnhum.2013.00479 23986677 PMC3752525

[B93] Power J. D. Cohen A. L. Nelson S. M. Wig G. S. Barnes K. A. Church J. A.. (2011). Functional network organization of the human brain. Neuron 72, 665–678. 10.1016/j.neuron.2011.09.006 22099467 PMC3222858

[B94] Preti M. G. Bolton T. A. Van De Ville D. (2017). The dynamic functional connectome: State-of-the-art and perspectives. Neuroimage 160, 41–54. 10.1016/j.neuroimage.2016.12.061 28034766

[B95] Preti M. G. Van De Ville D. (2017). Dynamics of functional connectivity at high spatial resolution reveal long-range interactions and fine-scale organization. Sci. Rep. 7:12773. 10.1038/s41598-017-12993-1 28986564 PMC5630612

[B96] Qin J. Chen S. G. Hu D. Zeng L. L. Fan Y. M. Chen X. P.. (2015). Predicting individual brain maturity using dynamic functional connectivity. Front. Hum. Neurosci. 9:418. 10.3389/fnhum.2015.00418 26236224 PMC4503925

[B97] Qiu L. Xia M. Cheng B. Yuan L. Kuang W. Bi F. (2018). Abnormal dynamic functional connectivity of amygdalar subregions in untreated patients with first-episode major depressive disorder. J. Psychiatry Neurosci. 43, 262–272. 10.1503/jpn.170112 29947609 PMC6019355

[B98] Quevenco F. C. Preti M. G. van Bergen J. M. Hua J. Wyss M. Li X.. (2017). Memory performance-related dynamic brain connectivity indicates pathological burden and genetic risk for Alzheimer's disease. Alzheimers Res. Ther. 9:24. 10.1186/s13195-017-0249-7 28359293 PMC5374623

[B99] Rashid B. Arbabshirani M. R. Damaraju E. Cetin M. S. Miller R. Pearlson G. D.. (2016). Classification of schizophrenia and bipolar patients using static and dynamic resting-state fMRI brain connectivity. Neuroimage 134, 645–657. 10.1016/j.neuroimage.2016.04.051 27118088 PMC4912868

[B100] Rashid B. Blanken L. M. E. Muetzel R. L. Miller R. Damaraju E. Arbabshirani M. R.. (2018a). Connectivity dynamics in typical development and its relationship to autistic traits and autism spectrum disorder. Hum. Brain Mapp. 39, 3127–3142. 10.1002/hbm.24064 29602272 PMC6045960

[B101] Rashid B. Chen J. Rashid I. Damaraju E. Liu J. Miller R.. (2018b). A framework for linking resting-state chronnectome/genome features in schizophrenia: a pilot study. Neuroimage 184, 843–854. 10.1016/j.neuroimage.2018.10.004 30300752 PMC6230505

[B102] Rashid B. Damaraju E. Pearlson G. D. Calhoun V. D. (2014). Dynamic connectivity states estimated from resting fMRI Identify differences among Schizophrenia, bipolar disorder, and healthy control subjects. Front. Hum. Neurosci. 8:897. 10.3389/fnhum.2014.00897 25426048 PMC4224100

[B103] Ridley B. Wirsich J. Bettus G. Rodionov R. Murta T. Chaudhary U. (2017). Simultaneous intracranial EEG-fMRI shows inter-modality correlation in time-resolved connectivity within normal areas but not within epileptic regions. Brain Topogr. 30, 639–655. 10.1007/s10548-017-0551-5 28194612

[B104] Rocca M. Hidalgo de la Cruz M. Valsasina P. Mesaros S. Martinovic V. Ivanovic J.. (2019). Two-year dynamic functional network connectivity in clinically isolated syndrome. Mult. Scler. 10.1177/1352458519837704. [Epub ahead of print]. 30887875

[B105] Rocca M. A. Valsasina P. Absinta M. Riccitelli G. Rodegher M. E. Misci P.. (2010). Default-mode network dysfunction and cognitive impairment in progressive MS. Neurology 74, 1252–1259. 10.1212/WNL.0b013e3181d9ed91 20404306

[B106] Rocca M. A. Valsasina P. Leavitt V. M. Rodegher M. Radaelli M. Riccitelli G. C.. (2018). Functional network connectivity abnormalities in multiple sclerosis: correlations with disability and cognitive impairment. Mult. Scler. 24, 459–471. 10.1177/1352458517699875 28294693

[B107] Rocca M. A. Valsasina P. Martinelli V. Misci P. Falini A. Comi G.. (2012). Large-scale neuronal network dysfunction in relapsing-remitting multiple sclerosis. Neurology 79, 1449–1457. 10.1212/WNL.0b013e31826d5f10 22955126

[B108] Roosendaal S. D. Schoonheim M. M. Hulst H. E. Sanz-Arigita E. J. Smith S. M. Geurts J. J.. (2010). Resting state networks change in clinically isolated syndrome. Brain 133(Pt 6), 1612–1621. 10.1093/brain/awq058 20356855

[B109] Rubinov M. Sporns O. (2010). Complex network measures of brain connectivity: uses and interpretations. Neuroimage 52, 1059–1069. 10.1016/j.neuroimage.2009.10.003 19819337

[B110] Ryyppo E. Glerean E. Brattico E. Saramaki J. Korhonen O. (2018). Regions of Interest as nodes of dynamic functional brain networks. Netw. Neurosci. 2, 513–535. 10.1162/netn_a_00047 30294707 PMC6147715

[B111] Sakoglu U. Pearlson G. D. Kiehl K. A. Wang Y. M. Michael A. M. Calhoun V. D. (2010). A method for evaluating dynamic functional network connectivity and task-modulation: application to schizophrenia. Magma 23, 351–366. 10.1007/s10334-010-0197-8 20162320 PMC2891285

[B112] Sandler S. I. (2006). Chemical, Biochemical and Engineering Thermodynamics, 4th Edn. Hoboken, NJ: John Wiley & Sons, Inc.

[B113] Sbardella E. Petsas N. Tona F. Pantano P. (2015). Resting-State fMRI in MS: general concepts and brief overview of its application. Biomed Res. Int. 2015:212693. 10.1155/2015/212693 26413509 PMC4564590

[B114] Schoonheim M. M. Hulst H. E. Brandt R. B. Strik M. Wink A. M. Uitdehaag B. M.. (2015). Thalamus structure and function determine severity of cognitive impairment in multiple sclerosis. Neurology 84, 776–783. 10.1212/WNL.0000000000001285 25616483

[B115] Shen H. Li Z. Qin J. Liu Q. Wang L. Zeng L. L.. (2016). Changes in functional connectivity dynamics associated with vigilance network in taxi drivers. Neuroimage 124(Pt A), 367–378. 10.1016/j.neuroimage.2015.09.010 26363345

[B116] Shi L. Sun J. Wu X. Wei D. Chen Q. Yang W.. (2018). Brain networks of happiness: dynamic functional connectivity among the default, cognitive and salience networks relates to subjective well-being. Soc. Cogn. Affect. Neurosci. 13, 851–862. 10.1093/scan/nsy059 30016499 PMC6123521

[B117] Shirer W. R. Ryali S. Rykhlevskaia E. Menon V. Greicius M. D. (2012). Decoding subject-driven cognitive states with whole-brain connectivity patterns. Cereb. Cortex 22, 158–165. 10.1093/cercor/bhr099 21616982 PMC3236795

[B118] Smith D. M. Zhao Y. Keilholz S. D. Schumacher E. H. (2018). Investigating the intersession reliability of dynamic brain-state properties. Brain Connect. 8, 255–267. 10.1089/brain.2017.0571 29924644 PMC6909700

[B119] Sun Y. Collinson S. L. Suckling J. Sim K. (2018). Dynamic reorganization of functional connectivity reveals abnormal temporal efficiency in schizophrenia. Schizophr. Bull. 45, 659–669. 10.1093/schbul/sby077 PMC6483577 29878254

[B120] Syed M. F. Lindquist M. A. Pillai J. J. Agarwal S. Gujar S. K. Choe A. S.. (2017). Dynamic functional connectivity states between the dorsal and ventral sensorimotor networks revealed by dynamic conditional correlation analysis of resting-state functional magnetic resonance imaging. Brain Connect. 7, 635–642. 10.1089/brain.2017.0533 28969437

[B121] Tagliazucchi E. Balenzuela P. Fraiman D. Chialvo D. R. (2012). Criticality in large-scale brain FMRI dynamics unveiled by a novel point process analysis. Front. Physiol. 3:15. 10.3389/fphys.2012.00015 22347863 PMC3274757

[B122] Tagliazucchi E. van Someren E. J. W. (2017). The large-scale functional connectivity correlates of consciousness and arousal during the healthy and pathological human sleep cycle. Neuroimage 160, 55–72. 10.1016/j.neuroimage.2017.06.026 28619656

[B123] Tagliazucchi E. von Wegner F. Morzelewski A. Brodbeck V. Jahnke K. Laufs H. (2013). Breakdown of long-range temporal dependence in default mode and attention networks during deep sleep. Proc. Natl. Acad. Sci. U.S.A. 110, 15419–15424. 10.1073/pnas.1312848110 24003146 PMC3780893

[B124] Tona F. Petsas N. Sbardella E. Prosperini L. Carmellini M. Pozzilli C.. (2014). Multiple sclerosis: altered thalamic resting-state functional connectivity and its effect on cognitive function. Radiology 271, 814–821. 10.1148/radiol.14131688 24484065

[B125] Tzourio-Mazoyer N. Landeau B. Papathanassiou D. Crivello F. Etard O. Delcroix N.. (2002). Automated anatomical labeling of activations in SPM using a macroscopic anatomical parcellation of the MNI MRI single-subject brain. Neuroimage 15, 273–289. 10.1006/nimg.2001.0978 11771995

[B126] van Geest Q. Douw L. van 't Klooster S. Leurs C. E. Genova H. M. Wylie G. R.. (2018a). Information processing speed in multiple sclerosis: Relevance of default mode network dynamics. Neuroimage Clin. 19, 507–515. 10.1016/j.nicl.2018.05.015 29984159 PMC6030565

[B127] van Geest Q. Hulst H. E. Meijer K. A. Hoyng L. Geurts J. J. G. Douw L. (2018b). The importance of hippocampal dynamic connectivity in explaining memory function in multiple sclerosis. Brain Behav. 8:e00954. 10.1002/brb3.954 29761008 PMC5943730

[B128] Vergara V. M. Mayer A. R. Damaraju E. Kiehl K. A. Calhoun V. (2017). Detection of mild traumatic brain injury by machine learning classification using resting state functional network connectivity and fractional anisotropy. J. Neurotrauma 34, 1045–1053. 10.1089/neu.2016.4526 27676221 PMC5333571

[B129] Vergara V. M. Mayer A. R. Kiehl K. A. Calhoun V. D. (2018). Dynamic functional network connectivity discriminates mild traumatic brain injury through machine learning. Neuroimage Clin. 19, 30–37. 10.1016/j.nicl.2018.03.017 30034999 PMC6051314

[B130] Vidaurre D. Abeysuriya R. Becker R. Quinn A. J. Alfaro-Almagro F. Smith S. M.. (2018). Discovering dynamic brain networks from big data in rest and task. Neuroimage 180(Pt B), 646–656. 10.1016/j.neuroimage.2017.06.077 28669905 PMC6138951

[B131] Wang Y. Berglund I. S. Uppman M. Li T. Q. (2018). Juvenile myoclonic epilepsy has hyper dynamic functional connectivity in the dorsolateral frontal cortex. Neuroimage Clin. 21:101604. 10.1016/j.nicl.2018.11.014 30527355 PMC6412974

[B132] Wang Z. Li Y. Childress A. R. Detre J. A. (2014). Brain entropy mapping using fMRI. PLoS ONE 9:e89948 10.1371/journal.pone.0089948 24657999 PMC3962327

[B133] Weiner M. W. Veitch D. P. Aisen P. S. Beckett L. A. Cairns N. J. Green R. C.. (2017). Recent publications from the Alzheimer's disease neuroimaging initiative: reviewing progress toward improved AD clinical trials. Alzheimers Dement. 13, e1–e85. 10.1016/j.jalz.2016.11.007 28342697 PMC6818723

[B134] Xu H. Su J. Qin J. Li M. Zeng L. L. Hu D.. (2018). Impact of global signal regression on characterizing dynamic functional connectivity and brain states. Neuroimage 173, 127–145. 10.1016/j.neuroimage.2018.02.036 29476914

[B135] Yaesoubi M. Allen E. A. Miller R. L. Calhoun V. D. (2015a). Dynamic coherence analysis of resting fMRI data to jointly capture state-based phase, frequency, and time-domain information. Neuroimage 120, 133–142. 10.1016/j.neuroimage.2015.07.002 26162552 PMC4589498

[B136] Yaesoubi M. Miller R. L. Bustillo J. Lim K. O. Vaidya J. Calhoun V. D. (2017a). A joint time-frequency analysis of resting-state functional connectivity reveals novel patterns of connectivity shared between or unique to schizophrenia patients and healthy controls. Neuroimage Clin. 15, 761–768. 10.1016/j.nicl.2017.06.023 28706851 PMC5496209

[B137] Yaesoubi M. Miller R. L. Calhoun V. D. (2015b). Mutually temporally independent connectivity patterns: a new framework to study the dynamics of brain connectivity at rest with application to explain group difference based on gender. Neuroimage 107, 85–94. 10.1016/j.neuroimage.2014.11.054 25485713 PMC4631126

[B138] Yaesoubi M. Miller R. L. Calhoun V. D. (2017b). Time-varying spectral power of resting-state fMRI networks reveal cross-frequency dependence in dynamic connectivity. PLoS ONE 12:e0171647. 10.1371/journal.pone.0171647 28192457 PMC5305250

[B139] Yang Z. Craddock R. C. Margulies D. S. Yan C. G. Milham M. P. (2014). Common intrinsic connectivity states among posteromedial cortex subdivisions: Insights from analysis of temporal dynamics. Neuroimage 93(Pt 1), 124–137. 10.1016/j.neuroimage.2014.02.014 24560717 PMC4010223

[B140] Yeo B. T. Krienen F. M. Sepulcre J. Sabuncu M. R. Lashkari D. Hollinshead M.. (2011). The organization of the human cerebral cortex estimated by intrinsic functional connectivity. J. Neurophysiol. 106, 1125–1165. 10.1152/jn.00338.2011 21653723 PMC3174820

[B141] Yu Q. Erhardt E. B. Sui J. Du Y. He H. Hjelm D.. (2015). Assessing dynamic brain graphs of time-varying connectivity in fMRI data: application to healthy controls and patients with schizophrenia. Neuroimage 107, 345–355. 10.1016/j.neuroimage.2014.12.020 25514514 PMC4300250

[B142] Yue J. L. Li P. Shi L. Lin X. Sun H. Q. Lu L. (2018). Enhanced temporal variability of amygdala-frontal functional connectivity in patients with schizophrenia. Neuroimage Clin. 18, 527–532. 10.1016/j.nicl.2018.02.025 29560309 PMC5857898

[B143] Zalesky A. Breakspear M. (2015). Towards a statistical test for functional connectivity dynamics. Neuroimage 114, 466–470. 10.1016/j.neuroimage.2015.03.047 25818688

[B144] Zalesky A. Fornito A. Bullmore E. T. (2010). Network-based statistic: identifying differences in brain networks. Neuroimage 53, 1197–1207. 10.1016/j.neuroimage.2010.06.041 20600983

[B145] Zalesky A. Fornito A. Cocchi L. Gollo L. L. Breakspear M. (2014). Time-resolved resting-state brain networks. Proc. Natl. Acad. Sci. U.S.A. 111, 10341–10346. 10.1073/pnas.1400181111 24982140 PMC4104861

[B146] Zhang C. Baum S. A. Adduru V. R. Biswal B. B. Michael A. M. (2018). Test-retest reliability of dynamic functional connectivity in resting state fMRI. Neuroimage 183, 907–918. 10.1016/j.neuroimage.2018.08.021 30120987

[B147] Zhang W. Li S. Wang X. Gong Y. Yao L. Xiao Y.. (2018). Abnormal dynamic functional connectivity between speech and auditory areas in schizophrenia patients with auditory hallucinations. Neuroimage Clin. 19, 918–924. 10.1016/j.nicl.2018.06.018 30003029 PMC6039841

[B148] Zhi D. Calhoun V. D. Lv L. Ma X. Ke Q. Fu Z.. (2018). Aberrant dynamic functional network connectivity and graph properties in major depressive disorder. Front. Psychiatry 9:339. 10.3389/fpsyt.2018.00339 30108526 PMC6080590

[B149] Zhou F. Zhuang Y. Gong H. Zhan J. Grossman M. Wang Z. (2016). Resting state brain entropy alterations in relapsing remitting multiple sclerosis. PLoS ONE 11:e0146080. 10.1371/journal.pone.0146080 26727514 PMC4699711

[B150] Zhou J. Liu S. Ng K. K. Wang J. (2017). Applications of resting-state functional connectivity to neurodegenerative disease. Neuroimaging Clin. N. Am. 27, 663–683. 10.1016/j.nic.2017.06.007 28985936

[B151] Zilles K. Amunts K. (2010). Centenary of Brodmann's map–conception and fate. Nat. Rev. Neurosci. 11, 139–145. 10.1038/nrn2776 20046193

